# Linear angular momentum multiplexing—conceptualization and experimental evaluation with antenna arrays

**DOI:** 10.1098/rspa.2020.0209

**Published:** 2020-10-07

**Authors:** Tim W. C. Brown, Ben H. Allen, Timothy D. Drysdale, Upasana K. Dagia

**Affiliations:** 1 Institute for Communication, Systems, University of Surrey, Guildford GU2 7XH, UK; 2 Department of Engineering Science, University of Oxford, Parks Road, Oxford OX1 3PJ, UK; 3 Network Rail, The Quadrant:MK, Elder Gate, Milton Keynes MK9 1ER, UK; 4 Institute for Digital Communications, University of Edinburgh, Edinburgh EH9 3FG, UK

**Keywords:** wireless communications, orbital angular momentum multiplexing, linear angular momentum multiplexing, MIMO

## Abstract

Linear angular momentum multiplexing is a new method for providing highly spectrally efficient short-range communication between a transmitter and receiver, where one may move at speed transverse to the propagation. Such applications include rail, vehicle and hyperloop transport systems communicating with fixed infrastructure on the ground. This paper describes how the scientific concept of linear angular momentum multiplexing evolves from orbital angular momentum multiplexing. The essential parameters for implementing this concept are a long array at least at one of the ends of the link; antenna element radiation characteristics and the array element spacing relative to the propagation distance. These parameters are also backed by short-range measurements carried out at 2.4 GHz used to model the Rice fading channel and determine resilience to multipath fading.

## 1. Introduction

Antenna arrays have historically served two main purposes in wireless communication. First, as diversity antennas to mitigate multipath fading in non line-of-sight environments [1] and second as beamforming arrays that reduce the path loss in line-of-sight links [2]. These techniques overcome the effects of multipath fading or path loss in order to improve the received signal-to-noise ratio level and subsequently reduce the digital symbol error rate at the receiver. Within the last two decades, diversity systems have further evolved into a new class of multiple-input multiple-output (MIMO) links that exploit the non-line-of-sight channels to increase the data throughput for a given frequency bandwidth [3]. Such systems are highly successful in spectrally congested radio environments but suffer a performance reduction in line-of-sight environments. Limited improvements are possible via spatial multiplexing, at the cost of large antenna spacing [4], or via polarization multiplexing using linear [5,6], circular [7] or even elliptical polarization [8] to produce two independent channels. These improvements are not sufficient to achieve parity of data throughput for mobile digital radio users both when they are in a city, and further afield such as travelling on a mass-transit system outside of an urban environment. Hence, further improvement to the data throughput in line-of-sight links is required.

Recent work has focused on generating spatial electromagnetic modes with independent phase patterns, so that signals can be transmitted in the same space and at the same frequency with low crosstalk. Originally explored in the context of optical communications, spectroscopy and optical tweezing, orbital angular momentum (OAM) modes have attracted increasing interest in the radio community since a demonstration nearly a decade ago [9], that built on earlier work to translate OAM into the radio domain [10,11]. OAM multiplexing relies on the angular change in signal phase around the plane that is transverse to the direction of propagation. The phase increases (or decreases) with angle around the direction of propagation, with a gradient that is an integer multiple of 2π radians. Different gradients correspond to different independent modes that permit greater data throughput than by multiplexing using polarization alone. Hence OAM has been experimented with for its capability to work in fixed line-of-sight communication links [12–14], using a variety of techniques such as circular arrays [15–18], reflectarray and transmitarray metasurfaces [19–24].

The major drawback of OAM modes is a diverging boresight null that is so pronounced that, in a naive implementation, the path loss as a function of antenna separation *d* approaches *d*^−4^, which is a performance typical of a near-field system (even though the underlying Laguerre-Gaussian modes are well known to be propagating modes [25]). The longer the link, the larger the antenna arrays that are required. Techniques such as modifying the array elements [15] and applying lens correction at the transmitter [26] can help to alleviate this. Nevertheless, the applications of OAM in radio appear to be limited to short-range fixed wireless links of tens of metres, such as would be required in backhauling links. On the other hand, OAM radio modes are not well suited to close-range communications (less than 2 m) when there is the potential for misalignment between the endpoints, such as would be the case for a moving vehicle. Therefore, for transport systems where one antenna is moving at speed on a vessel transverse to the direction of propagation while the other is fixed on the ground at the side, an alternate multiplexing technique is required.

This paper describes just such a technique, which evolves from OAM multiplexing to consider linear angular momentum (LAM) multiplexing. This concept unwraps the OAM modes into their linear equivalent. LAM multiplexing further exploits the relative lateral translation of the transmit and receive antennas (perpendicular to the link) to create the large effective aperture that is needed to distinguish between modes; analogously to other synthetic aperture techniques, the effective aperture is extended in the direction of travel, allowing a relatively small-receive antenna to sample the spatially distributed phase patterns of each of the independent LAM modes. In this way, for the special case of the vehicular geometries considered, LAM multiplexing achieves similar capacity enhancements to OAM multiplexing, but does so using low-profile antenna elements and overall array dimensions that are consistent with application to the ground to rail, hyperloop or vehicle links as examples [27]. It is worthy of noting that LAM multiplexing may be thought of as like that of near-field MIMO [28], which is rather designed for static links in the near field.

This paper presents for the first time a rigorous analysis of the LAM multiplexing principle (in §2) and elucidates the implementation requirements for the predominant form of application (linear array antennas, in §3). These parameters include inter-element spacing required; antenna characteristics and frequency bands which could be chosen. A low-profile implementation of LAM suitable for a moving vessel is shown in §4. In §5, measurements that characterize the short-range propagation channel are reported, which discuss the resilience of LAM to self-reflections (the dominant fading mechanism in LAM deployments). Section 6 concludes the paper.

## 2. Definition of LAM modes

**Figure 1.**
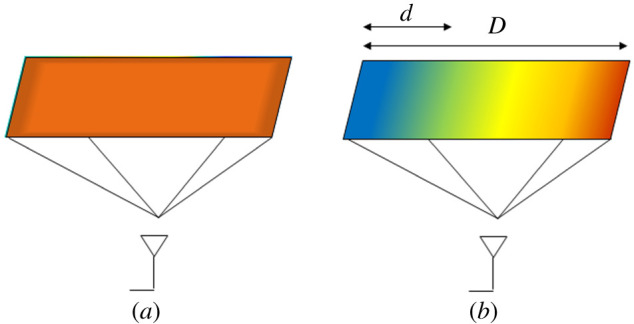
Heat map illustration of the (*a*) constant magnitude distribution and (*b*) linearly changing phase distribution over the plane of a *l* = +1 LAM mode. (Online version in colour.)

**Figure 2.**
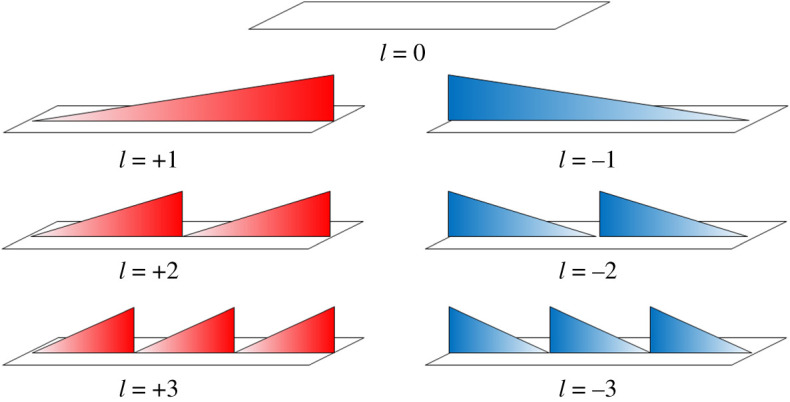
Illustration of LAM modes for *l* = −3 to 3. (Online version in colour.)

It is useful to illustrate the LAM modes where figures 1 and 2 are analogous to how OAM modes are illustrated in [29]. Figure 1*a* shows an example plane where there is a uniform magnitude distribution for a LAM mode. Figure 1*b* shows its corresponding phase changing linearly from 0 to 2π in this case.

The modes are defined by integer *l*, which for LAM is used to find the phase *ψ* corresponding to distance *d* along the plane that has total length *D* shown in figure 1*b* thus:

2.1 $$\psi =l\frac{d}{D}2\pi .$$

The modes from *l* = −3 to +3 are illustrated in figure 2, which correspond to a total number of modes*, N* = 6 in this case as |*l*| cannot exceed *N*/2. Increasing positive values of *l* correspond to an increasing positive phase gradient, while negative values correspond to an increasing negative gradient. The phase ranges from a minimum of zero to a maximum of 2π radians. There is no phase change for *l* = 0, which exhibits a constant zero phase. Implementation of LAM requires an antenna, which can vary the phase along a linear plane and maintain magnitude. Such an example is a tapered dielectric lens backed with a waveguide cavity, though many implementations are possible.

**Figure 3.**
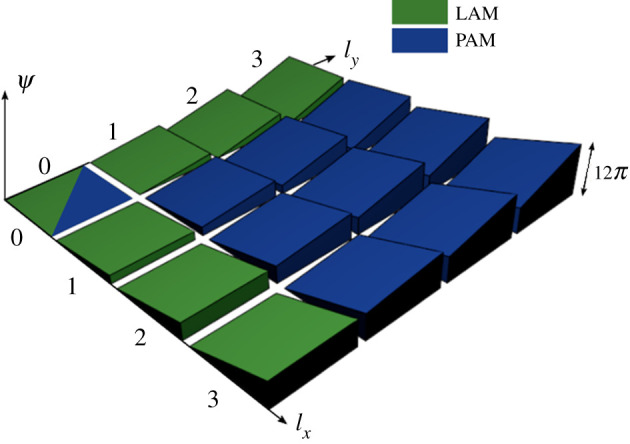
Three-dimensional heat map representation of the phase gradients for planar angular momentum with modes *l* = (0,0) through to *l* = (3,3). (Online version in colour.)

Planar angular momentum (PAM) is also proposed, where the phase gradient varies in the *x* direction, as with LAM, and in the *y* direction also. In a PAM implementation, the modes must be considered in the coordinate form (*l_x_*,*l_y_*) whereby neither |*l_x_*| nor |*l_y_*| must exceed *N*/4, while *N* ≥ 4 is a minimum requirement such that *N* is an even integer. While |*l*_max_| is a maximum value of |*l_x_*| and |*l_y_*|, then *N* = 4|*l*_max_| or *N* = 4|*l*_max_| + 2 are two possible solutions. The positive modes for a PAM deployment, with |*l*_max_| = 3 and hence *N* = 12 or 14, are illustrated in figure 3, where the negative gradient modes are excluded for clarity. Though figure 3 could be expanded to 49, or (2|*l*_max_| + 1)^2^ in general, visual modes if all −*l_x_* and −*l_y_* modes were included, only *N* of these modes are orthogonal to each other, while the remaining are degenerative in that they would not maintain orthogonality. Also indicated in figure 3 by showing the green modes, an *N* = 6 LAM is a subset of this PAM example where it can operate in the *x* direction with modes −3 ≤ *l_x_* ≤ 3 and in the *y* direction with −3 ≤ *l_y_* ≤ 3. In this paper, PAM is deemed not within the scope and will not be studied further. However, for reference purposes to any future work, the mathematical definitions of PAM are detailed in the appendix.

As with OAM, a simpler method is to implement LAM in discrete form by creating a linear array of antenna elements or to use a leaky wave antenna, which are within the scope of the work in this paper. Therefore, equation (2.1) is modified to the following where element *n* is at a discrete displacement point *d_n_*:

2.2 $$\psi (n)=l\frac{d_{n}}{D}2\pi =l\frac{n}{N}2\pi .$$

**Figure 4.**
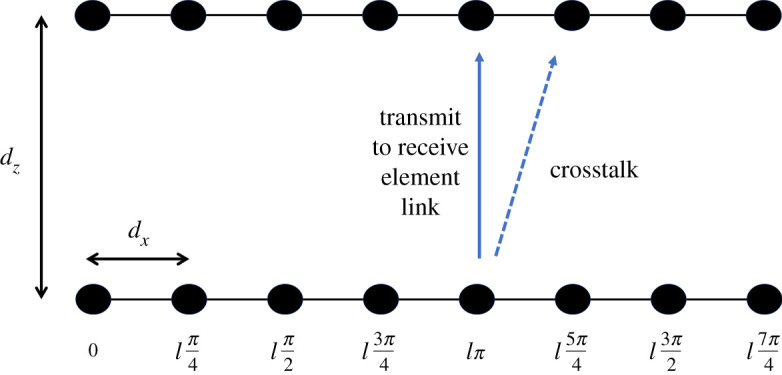
Illustration of the set-up for detection of LAM modes *l* = −4 to *l* = +4 for an *N* = 8 linear array. (Online version in colour.)

In the far field, the maximum OAM mode number that can be generated using an array of *N* elements is bound by the criteria that |*l*| < *N*/2 [10]. Therefore, in general, there are *N *− 2 OAM modes available if the zero mode, *l* = 0, is ignored. In the case where |*l*| = *N*/2, there are an insufficient number of elements to construct the OAM phase pattern at the boresight in the far field and the Laguerre-Gauss (LG) beam does not properly form [17]. This is seen by inspection of equation (2.2), that when |*l*| = *N*/2, the phase for each element will alternate between 0 and *π* radians as *n* is incremented, giving two degenerative modes, which will cause the phase pattern to fail. However, the |*l*| < *N*/2 limitation on the number of modes generated is based on two assumptions. The first is that each element has a wide beamwidth and second that the transmit-to-receive distance is in the far field. If each element in the uniform circular array for OAM was highly directive, i.e. with a narrow beamwidth, it would become too difficult for the individual patterns to superimpose and form an array pattern with an LG beam. This would be further exacerbated as the radius of the array increases with narrow beams. Also, as found in [30], when the transmit and receive circular arrays are separated close to, or nearer than the Rayleigh distance based on their diameters, then the transmit and receive elements can readily couple to each other and the fundamental capacity limit is nearly reached similar to a scenario of near-field MIMO [28]. Therefore, either with directive element beams or coupling in the near field, all available OAM modes are fully exploited. These two principles applicable to OAM can be readily applied to LAM, which can be analysed in figure 4 illustrating an *N *= 8 LAM link with transmit to receive distance *d_z_* and element separation *d_x_*.

There are two ways the eight modes in figure 4, namely *l* = 0; ±1; ±2; ±3 for the first seven modes and one of the two degenerative modes −4 or +4 for the eighth mode, can operate. They are as follows and will be evaluated in §3:

(1) By maintaining a high enough ratio *d_x_*/*d_z_*, which requires short transmit to receive distance to be practical. If the ratio is too low, only mode *l* = 0 would work and it would become a beamforming rich channel [3]. Array patterns would form at both ends based on the well-known array factor [31].
(2) For a given spacing *d_x_*, the beamwidth of each element's radiation pattern needs to be narrow enough, or the pattern needs to be directive enough, so as to minimize crosstalk as illustrated with the fifth and sixth elements in figure 4. High enough directivity and *d_x_*/*d_z_* ratio can principally exhibit zero crosstalk that falls below the receiver noise floor dependent on system noise temperature and bandwidth.

**Figure 5.**
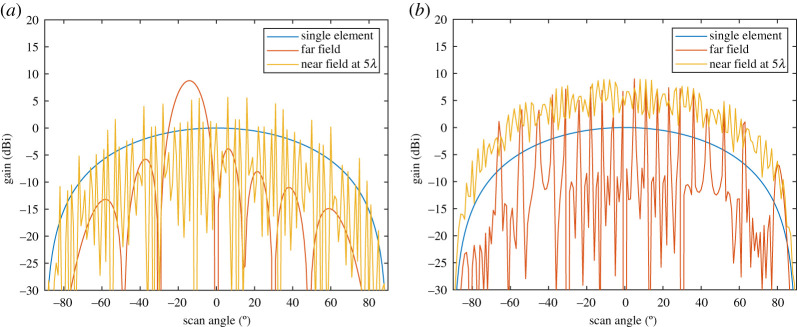
Comparison of single element and *N* = 8 element array transmitting mode *l* = −1 synthesized in the near field and far field for (*a*) half wavelength and (*b*) ten wavelength element spacing *d_x_*. (Online version in colour.)

A further point to note in terms of the short range of a LAM link is the definition of the far field, which can be interpreted in two ways. One interpretation would be that of the whole array, and hence the Rayleigh distance [30] can be derived as 2(*d_x_*(*N *− 1))^2^/λ, where λ is the free space wavelength of the frequency of operation. This far field definition could be used to determine the maximum value of *d_z_* at which all *N* modes are functional. If this distance is exceeded, the far field pattern of the array will form. Two examples of this are given in figure 5 when *N* = 8 by plotting the absolute gain patterns for (a) *d_x_* = λ/2 and (b) *d_x_* = 10λ when *l* = −1, and it is assumed each element has a peak gain of 0 dBi as shown. In both cases, the far field array gain is negligible below −30 dBi at boresight, just as would occur in OAM as the phases in the elements add in a deconstructive way, which causes rapid loss in link capacity for high transmit to receive distance [30]. Also shown in figure 5*a* and *b* are the near-field patterns when *d_z_* is equal to 5λ below the array's Rayleigh distance. Clearly, the antiphase nulls below −30dBi and grating lobe effects are avoided when in the near field, which will help LAM transmission; but at the same time, there is a ‘fading' created on the antenna pattern. At the boresight, which is the region of interest, this causes a lower peak gain than what can be achieved in the far field, but for 10λ it can be seen in figure 5*b* that the drop is lower than 3 dB, which is acceptable. Another interpretation of Rayleigh distance, particularly when *d_x_* is large, is to consider the maximum dimension of an element, *D*_Elem_. With widely spaced elements that have a narrow enough beamwidth to cause negligible crosstalk, each element could be considered as independently radiating its own electromagnetic waves and therefore the Rayleigh distance becomes 2*D*_Elem_^2^/λ. As *D*_Elem_ ≪ *d_x_*(*N *− 1), this allows *d_z_* to be in the far field but at the same time it can readily support *N* modes. Based on defining the far field with *D*_Elem_, the dependency that element beamwidth and the *d_x_*/*d_z_* ratio have in order to reach the fundamental capacity limits are discussed in the next section. Furthermore, either the transmitter or receiver is moving in the ±*x* direction, which also needs to be taken into account.

## 3. Theoretical analysis of array dimensions for LAM multiplexing

There are two parts to the analysis in this section with regard to finding an effective LAM multiplexing link. First, to ensure that the full MIMO channel matrix rank can be achieved to obtain spatial multiplexing, which will be analysed in the first subsection. Second, the effect of offset when the elements are not facing each other will be analysed in the second subsection. These factors affect the choice of array spacing and antenna beamwidth.

### (a) Channel analysis with facing elements

**Figure 6.**
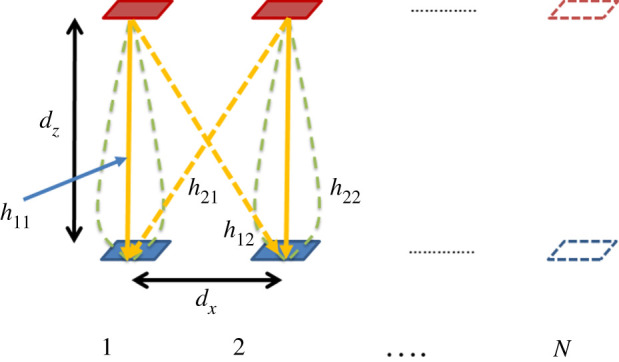
Channel coefficients for transmit and receive elements 1 and 2 in a LAM link with *N* modes. (Online version in colour.)

Figure 6 illustrates the four-channel matrix elements for the 2 × 2 LAM channel, **H**, with two transmit elements in red and two receive elements in blue. Coefficients *h*_11_ and *h*_22_ correspond to the aligned transmit-to-receive elements, while *h*_21_ and *h*_12_ correspond to crosstalk. Note that more transmit and receive elements could extend up to *N* but it is sufficient to analyse the effects of antenna element beamwidth and spacing with *N* = 2. Theoretically, the number of modes increases with *N*, though precision limitations in practice may limit the number of orthogonal modes as the ratio *n*/*N* in equation (2.2) would cause the phase increment between elements and subsequently modes to decrease.

It is assumed that the distance *d_z_* is beyond the element Rayleigh distance while it is also assumed all antenna elements are identical. With no multipath, a free space path loss model can be applied to determine the relative channel coefficients between elements using the antennas' relative amplitude pattern, *A*, in dB, which can correspond to an absolute maximum gain of 0 dBi at boresight defined by the following equation:

3.1 $$A(\theta_{t})\left(\mathrm{dB}\right)=\max[-180,20\log_{10}|\cos(k\theta_{t})|],$$

where *θ*_t_ is the angle relative to the boresight of the antenna element, while *k* can be found to correspond to a desired 3 dB beamwidth of the element. The channel **H** can be suitably set by applying the free space path loss equation [30] as follows:

3.2 $$\text{H}=\left(\begin{array}{cc} h_{11} & h_{21}\\ h_{12} & h_{22} \end{array}\right)=\frac{1}{N_{H}}\frac{\lambda }{4\pi }\left(\begin{array}{cc} \frac{e^{j\beta d_{z}}}{d_{z}} & \frac{A(\theta_{21})e^{j\beta \sqrt{d_{z}^{2}+d_{x}^{2}}}}{\sqrt{d_{z}^{2}+d_{x}^{2}}}\\ \frac{A(\theta_{12})e^{j\beta \sqrt{d_{z}^{2}+d_{x}^{2}}}}{\sqrt{d_{z}^{2}+d_{x}^{2}}} & \frac{e^{j\beta d_{z}}}{d_{z}} \end{array}\right)=\left(\begin{array}{cc} h_{C} & h_{X}\\ h_{X} & h_{C} \end{array}\right),$$

where *β* is the phase constant equal to 2π/λ. It should be noted that the angles *θ*_12_ = *θ*_21_ will be dependent upon *d_x_* and *d_z_* and result in two-channel coefficients: one for the transmit-to-receive element channel, *h*_C_, and the crosstalk channel, *h*_X_. The full singular value decomposition (SVD) matrix [32] can be computed to resolve the eigenvalues, λ_1_ and λ_2_, which are the square of the two singular values. The channel is normalized by factor *N*_H_ such that $\lambda_{1}^{2}$ and $\lambda_{2}^{2}=4$. It is noteworthy at this point that though wavelength, λ, is included in equation (3.2), the channel has minimal dependence on frequency or wavelength. Though |*h*_X_| has greater path loss than |*h*_C_|, the ratio of the two path losses is frequency independent and only affected by the relative difference in distances *d_z_* and √(*d_x_*^2^ + *d_z_*^2^) and the antenna radiation pattern. The relative phase between *h*_X_ and *h*_C_ is dependent on the same relative difference in distances but also frequency. However, as will be found, the frequency variation of the channel phase has minimal effect on LAM except where *d_x_*/*d_z_* is small, but does not normally occur. Therefore, the influence of frequency on LAM multiplexing can be ignored. The fundamental capacity limit, *C*_MIMO_, of the MIMO channel for a known signal-to-noise ratio, *ρ*/*σ*^2^, is resolved as follows where *ρ* is the signal power and *σ*^2^ is the noise power [32]:

3.3 $$C_{\mathrm{MIMO}}=\log_{2}\left(1+\frac{\rho }{2\sigma^{2}}\lambda_{1}\right)+\log_{2}\left(1+\frac{\rho }{2\sigma^{2}}\lambda_{2}\right).$$

The LAM discrete Fourier transform-based pre-coder uses the two modes, *l* = 0 and one of *l* = ±1, where *l* = +1 is arbitrarily chosen in this case and is applied as follows:

3.4 $${\text{H}}_{\mathrm{LAM}}=\frac{1}{\sqrt{2}}\left(\begin{array}{cc} 1 & 1\\ -1 & 1 \end{array}\right)\left(\begin{array}{cc} h_{C} & h_{X}\\ h_{X} & h_{C} \end{array}\right)\left(\begin{array}{cc} 1 & -1\\ 1 & 1 \end{array}\right)\frac{1}{\sqrt{2}}=\left(\begin{array}{cc} h_{C}+h_{X} & 0\\ 0 & h_{C}-h_{X} \end{array}\right).$$

Therefore, the pre-coded channel resolves two diagonal coefficients that are equal in magnitude to the singular values of the channel and therefore the eigenvalues are derived as λ_1_ = |*h*_C_ + *h*_X_|^2^ and λ_2_, = |*h*_C_ − *h*_X_|^2^. Note, however, that the LAM pre-coders are not necessarily equal to the singular vectors in SVD, and while the magnitude of the eigenvalues are the same as the square of the LAM pre-coder coefficients, they would have a different phase and are therefore complex numbers. This is in agreement with ([30] eq. (23)) whereby the LAM pre-coder is a unitary discrete Fourier transform matrix and the capacity limit for both LAM and SVD has no difference.

**Figure 7.**
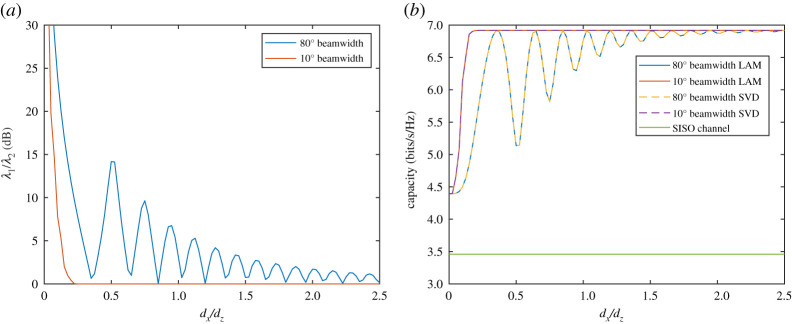
Plot of the 2 × 2 MIMO channel with (*a*) eigenvalue ratio and (*b*) capacity compared with SISO using both LAM and full SVD pre-coding with antenna beamwidths of 80^o^ and 10^o^. (Online version in colour.)

By inspection of figure 7, it can be seen that there are two conditions whereby the capacity limit is reached. Note that the capacity limits in figure 7*b* for SVD and LAM overlap as expected and 10 dB is chosen as an arbitrary signal-to-noise ratio that is used for all analysis in this paper. The first case for maximum capacity is when *h*_X_ has negligible or no magnitude. This occurs where the *d_x_*/*d_z_* ratio is high and the beamwidth is narrow, such as for a 10^o^ beamwidth where *d_x_*/*d_z_* > 1 showing the eigenvalue ratio to be 0 dB in figure 7*a* and then the capacity limit is reached. For wide beamwidths, however, the capacity limit can also be reached such as the case for an 80^o^ beamwidth when *d_x_*/*d_z_* is approximately 0.35 and 0.65. In these instances, the condition is met that arg(*h*_X_) = π/2 and |*h*_X_| ≈ |*h*_C_|, which meets criteria for full spatial multiplexing. It should be noted, however, that the points at which the peaks occur are dependent on frequency and in this instance, as with all analysis performed in this paper, λ = 1 m. Changing the frequency would alter the position of the peaks but the impact this has at large distances such as *d_x_*/*d_z_* > 1.5 is minimal because the capacity limit is nearly reached with low crosstalk. Therefore, it is best to use large spacing, *d_x_*, with low enough crosstalk to have minimal spatial multiplexing influence.

A final point to note about LAM multiplexing is that, for low ratios of *d_x_*/*d_z_* such as below 0.1 in figure 7, the capacity limit drops substantially. This is because in such cases the antenna spacing is too close such that the channels become highly correlated for the distance of propagation and it becomes a beamforming rich channel. Therefore, the capacity increases only as a result of an effective gain in signal-to-noise ratio of 3 dB at the receiver. Beyond this, full multiplexing is achieved with appropriate spacing and the capacity limit is twice that of the single-input single-output (SISO) channel as shown in figure 7*b*. It can be inferred that for higher values of *N* with the same beamwidth and spacing, capacity limits equal to *N* times the SISO capacity can be reached with *N* equal eigenvalues.

A final note to add to this subsection is to discuss the frequency range over which LAM multiplexing can operate. The only factor limiting the frequency that can be used is the Rayleigh distance based on the maximum element dimension, *D*_Elem_, assuming the antennas are spaced several wavelengths apart. This will normally be the case, which leaves the challenge to ensure the element size is sufficiently small thus resulting in a Rayleigh distance less than half the propagation distance, which can be assumed to be typically 0.5 m for practical applications. This means that when *D*_Elem_ = 0.5λ, it will allow far field propagation at the lowest frequency of 300 MHz. This opens the technology to virtually all radio spectra, from which a suitable frequency band could be chosen dictated by licensing and cost constraints. Larger element sizes such as *D*_Elem_ = λ would still have a wide range of spectra available, starting at 1.2 GHz. Increasing to 2λ would be more restrictive increasing the starting frequency to 4.8 GHz.

### (b) Channel analysis with offset elements

Analysis of the results presented in §3a show that, like OAM, LAM multiplexing does not bring anything conceptually new to radio communication as stated in [30], and the fundamental difference is that LAM creates the same multiplexing streams through the use of discrete Fourier transform-based pre-coders. This is computationally more efficient than SVD or other kinds of beamforming since the eigenmodes can be readily calculated at either the transmitter or receiver array, while the other end is completely passive in the sense that all elements are fixed with constant phase values. This benefit is only achieved through the appropriate topology of the array as well as the structural design of the antenna elements. The novel contribution in this work is to therefore focus on how array antennas for LAM can be deployed to enable highly spectrally efficient connectivity and high throughput short-range links while one end of the link is moving and that the simplicity of applying pre-coders is maintained, which is a capability unique to LAM.

**Figure 8.**
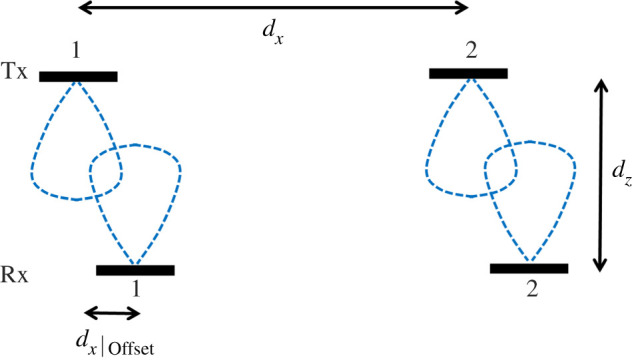
Dimensions of a 2 × 2 LAM channel from when offset in the *x* direction. (Online version in colour.)

To analyse the effect of mobility transverse to the direction of propagation, it is necessary to determine the effect of offsetting either the transmitter or receiver in the *x* direction by *d_x_*_|Offset_ as illustrated in figure 8. This will cause the LAM pre-coded capacity, *C*_LAM_, to be unequal to the capacity found by full SVD, which will also decrease as offset increases to halfway between the elements of the stationary array.

The channel with the introduction of the offset will cause the two crosstalk channels to become unequal and are calculated by updating equation (3.2) as follows noting that now *θ*_12_ ≠ *θ*_21_ when offset:

3.5 $$\begin{array}{r} {\text{H}}_{\mathrm{Offset}}=\frac{1}{N_{H}}\frac{\lambda }{4\pi }\left(\begin{array}{cc} \frac{e^{j\beta \sqrt{d_{z}^{2}+d_{x|\mathrm{Offset}}^{2}}}}{\sqrt{d_{z}^{2}+d_{x|\mathrm{Offset}}^{2}}} & \frac{A(\theta_{21})e^{j\beta \sqrt{d_{z}^{2}+{\left(d_{x}-d_{x|\mathrm{Offset}}\right)}^{2}}}}{\sqrt{d_{z}^{2}+{\left(d_{x}-d_{x|\mathrm{Offset}}\right)}^{2}}}\\ \frac{A(\theta_{12})e^{j\beta \sqrt{d_{z}^{2}+{\left(d_{x}+d_{x|\mathrm{Offset}}\right)}^{2}}}}{\sqrt{d_{z}^{2}+{\left(d_{x}+d_{x|\mathrm{Offset}}\right)}^{2}}} & \frac{e^{j\beta \sqrt{d_{z}^{2}+d_{x|\mathrm{Offset}}^{2}}}}{\sqrt{d_{z}^{2}+d_{x|\mathrm{Offset}}^{2}}} \end{array}\right)=\left(\begin{array}{cc} h_{\mathrm{Offset}|11} & h_{\mathrm{Offset}|21}\\ h_{\mathrm{Offset}|12} & h_{\mathrm{Offset}|22} \end{array}\right). \end{array}$$

When there is an offset, it is no longer possible to create two orthogonal eigenmodes with the discrete Fourier transform pre-coder so *h*_Offset|21_ and *h*_Offset|12_ become non-zero. This will form a signal-to-interference-plus-noise ratio (SINR) due to interference between the modes. The intermodal interference is calculated for the two modes as follows through which the capacity C_LAM_ can be derived:

3.6 $${\mathrm{SINR}}_{1}=\frac{{|h_{\mathrm{Offset}|11}|}^{2}}{{|h_{\mathrm{Offset}|21}|}^{2}+\sigma_{n}^{2}},\;{\mathrm{SINR}}_{2}=\frac{{|h_{\mathrm{Offset}|22}|}^{2}}{{|h_{\mathrm{Offset}|12}|}^{2}+\sigma_{n}^{2}}$$

and

3.7 $$C_{\mathrm{LAM}}=\log_{2}(1+{\mathrm{SINR}}_{1})+\log_{2}(1+{\mathrm{SINR}}_{2}).$$

**Figure 9.**
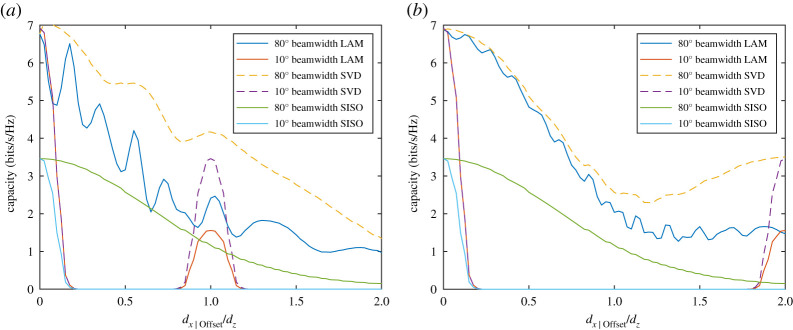
Plots showing a comparison of the MIMO capacity with LAM pre-coded capacity versus the ratio using antenna beamwidths of 80^o^ and 10^o^ versus *d_x_*_|Offset_/*d_z_* where (*a*) *d_x_*/*d_z_* = 1 and (*b*) *d_x_*/*d_z_* = 2. (Online version in colour.)

The capacity *C*_LAM_ is compared with *C*_MIMO_ in figure 9*a* for 10^o^ and 80^o^ beamwidths, where the element separation is set such that *d_x_*/*d_z_* = 1. This value is taken from figure 7*b*, over 90% of the maximum capacity limit is sufficiently reached for both beamwidths when *d_x_*/*d_z_* > 1. Taking the case where *d_x_*_|Offset_/*d_z_* = 1, *C*_MIMO_ becomes equal to the SISO capacity when there is no offset. This is because the channel *h*_Offset|21_ for both beamwidths is the only remaining channel while the other three are substantially lower in magnitude. The LAM pre-coded capacity, *C*_LAM_, has otherwise dropped to be significantly worse at this point since the modes are far from orthogonal causing high SINR. Starting from zero offset, *C*_MIMO_ drops to 90% of the maximum when *d_x_*_|Offset_/*d_z_* = 0.25, but conversely *C*_LAM_ oscillates substantially in this range when the beamwidth is 80^o^. There are two useful observations to make from this, first as the propagation channel is short range with minimal multipath fading, this means that LAM pre-coded channel can apply a phase correction to increase capacity towards *C*_MIMO_ when *d_x_*_|Offset_/*d_z_* is known. Second, the need for phase correction can otherwise be mitigated by using narrower beamwidth antennas, which would create higher SINR with LAM pre-coding. However, there is a clear trade off necessary as making the beamwidth too high, as seen from the 10^o^ beamwidth case, substantially limits the tolerable offset. Nonetheless, beamwidths in the range of 40^o^ to 60^o^ allow the offset effect to be tolerated. Another alternative for wide beamwidths is to increase the *d_x_*/*d_z_* ratio towards 2 where the same analysis is carried out in figure 9*b*. Here, it can be seen that *C*_LAM_ is comparable with *C*_MIMO_ up to *d_x_*_|Offset_/*d_z_* = 1 for the 80^o^ beamwidth. Nonetheless, the capacity drops to 90% of its maximum when *d_x_*_|Offset_/*d_z_* = 0.3 so therefore the resilience to offset has not improved substantially. This therefore indicates that to avoid a drop in capacity at large offsets, a new approach to the antenna designs is necessary to ensure seamless connectivity, where a solution is presented in the next section.

## 4. Low-profile antenna implementation of LAM multiplexing on a moving vessel

**Figure 10.**
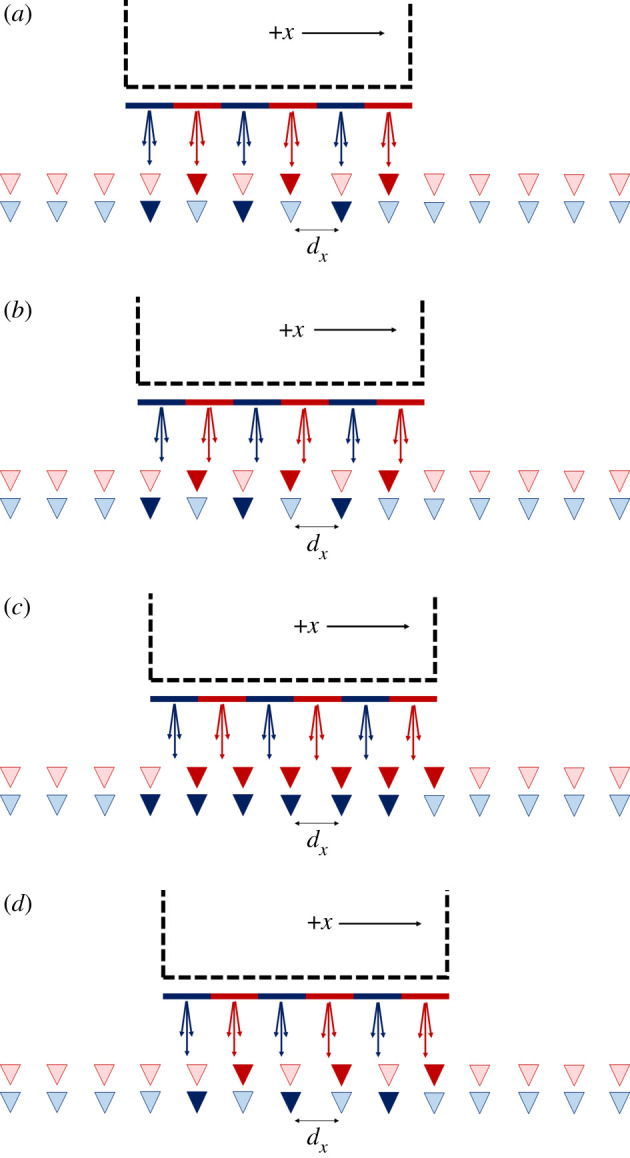
Snapshot illustrations of a moving vessel moving in the positive x direction when (*a*) transmit and receive antennas are initially facing, (*b*) moving antennas are partially offset, (*c*) transmit antennas are halfway between the gaps of the receivers and (*d*) offset the next step. (Online version in colour.)

In order to enable a seamless connection as the vessel is moving, a suitable concept is illustrated in figure 10, where it is assumed the receiver is fixed and the vessel transmitting. Only six transmit antennas are shown for purposes of clarity. It should be noted that this is an alternative concept compared with that in [27], which was designed more specifically for implementation underneath a train. The solution presented here could be applied more universally to the bottom, side or top of a moving vessel. Antennas in blue indicate they have polarization A, while antennas in red have polarization B whereby A and B are orthogonal. Adjacent antennas always alternate in polarization for purposes of substantially reducing crosstalk. Additionally, it should be noted all antennas have a high polarization purity. The receive antennas all have either a light or dark colour. This is to indicate when the antennas are illuminated and they become dark when this is the case. From the transmit antenna elements, the arrows in matching colours indicate that the antenna pattern is designed to have strong boresight gain but avoid crosstalk as the vessel is moving. Four snapshots are shown in figure 10: (*a*) where the antennas are facing; (*b*) then they are offset in the positive *x* direction but the same receive antennas are still illuminated; (*c*) a halfway point is reached where both adjacent receive antennas are illuminated and finally (*d*) they are offset to the next stage. This set-up indicates how, as the vessel is moving, a seamless connection can be obtained, while gaps at the transition point can be accommodated based on the analysis in §3b.

**Figure 11.**
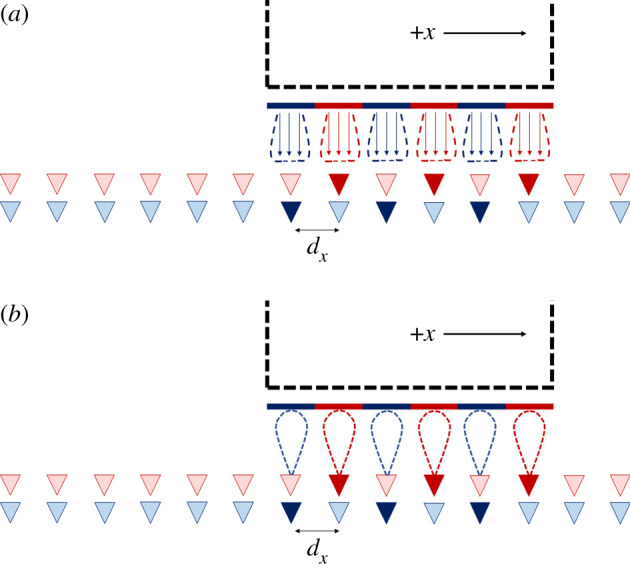
Illustration of the antenna patterns for (*a*) the transmitter on the moving vessel and (*b*) the receiver antennas. (Online version in colour.)

A final point to note, however, is the antenna patterns that would be required at the transmitter and receiver as shown in figure 11*a* and *b*, respectively. The transmitter antennas have a pattern such that at the receiver, their gain will not change as they are passing their corresponding receiver antennas. At the same time, the gain towards the direction of adjacent receiver antennas should be as low as possible to minimize crosstalk. Designing such an antenna element is a challenge, which may be achieved by a linear array of highly directive sub-elements that will not cause grating lobes, but such an arrangement would still cause ‘ripple' in the element beam due to a finite number of directive sub-element patterns. This would need to be minimized by choosing a suitable beamwidth for each element in the array. The use of alternating polarization will be of significant benefit to help reduce crosstalk. The corresponding receive antennas have a directive beam pattern, which will also assist in reducing crosstalk though the beamwidth would also need to be suitably wide to mitigate ripple.

## 5. Characterization of the propagation channel and evaluation of resilience to multipath

In the previous sections, the criteria for antenna spacings and antenna element patterns were established where the capacity limits derived are based upon assuming free space propagation between the transmit and receive elements. It is noteworthy that it is also analysed in the appendix that the phase error caused by Doppler shift of the line of sight rays is negligible that creates a strong resilience in LAM to fast-moving vessels. Multipath fading due to rays reflected off the ground or other surrounding objects, though they will be few, will have an effect that needs to be quantified. Therefore, it is necessary to model the first- and second-order statistics of the channel informed by measurements to characterize the strongly Ricean fading, also identified in [27], that will be expected due to a single dominant line-of-sight path. The Doppler spread [1], where the dominant line-of-sight component will have no shift, is normal to the direction of travel and is expected to have different multipath phase characteristics to traditional Rice channels, where its impact on capacity is mitigated when crosstalk is low.

### (a) Characterization of the propagation channel

**Figure 12.**
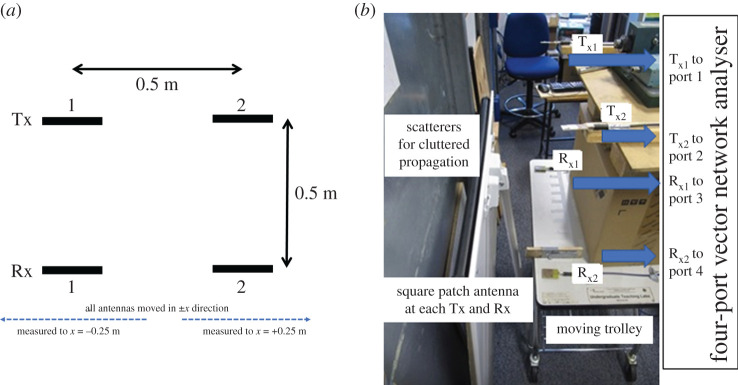
Illustration (*a*) of the measurement set-up and (*b*) a photograph of the test environment. (Online version in colour.)

A measurement set-up was carried out to characterize the propagation channel, whereby four simple square patch antennas as elements tuned to 2.4 GHz facing each other co-polarized, were aligned with a width and height of 0.5 m as shown in figure 12*a*. Each patch antenna had a 3 dB beamwidth of 80^o^ and this was purposely chosen so that they would be able to absorb as much multipath as possible while exhibiting worst case crosstalk when *d_x_*/*d_z_* = 1. The four antennas were situated in a cluttered laboratory environment as shown in figure 12*b* in order to create rich multipath to determine the lowest possible Rice factor that could be reached. All four parameters of the 2 × 2 channel **H** were measured by connecting the four patch antennas to a four-port vector network analyser with sufficient dynamic range. In order to create a fading channel, the moving trolley was offset from its initial position in 0.01 m steps in the ±*x* direction to a limit of ±0.25 m as shown in figure 12*a*. Therefore, 51 channel state samples were taken when all antennas were facing, which reflects a propagation scenario equivalent to that in figure 10. As the wavelength was 0.125 m, the sampling space of 0.01 m corresponds to a sampling frequency 12.5 times that of the maximum Doppler shift based on the phase gradient of the measurement.

**Figure 13.**
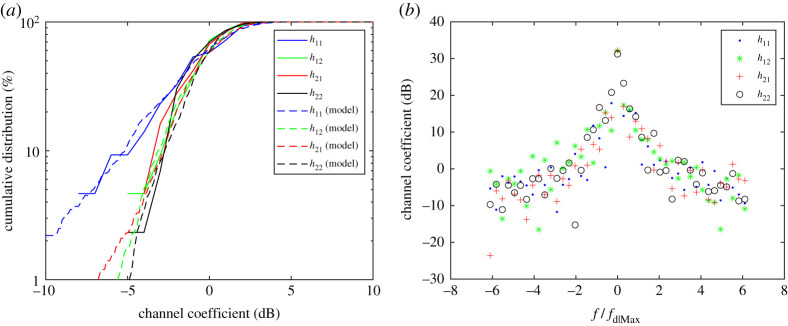
(*a*) Measurement results of the cumulative distribution of the channel coefficients compared with the Rice channel model (*b*) Measurement results of the corresponding Doppler spread. (Online version in colour.)

Figure 13*a* shows the cumulative distribution of the four measured channels as well as a modelled Rice channel with suitably fitting Rice factor. The resolved Rice factors are 4 for *h*_11_; 16 for *h*_22_; 11 for *h*_12_; and 9 for *h*_21_. Note that the crosstalk channels gave similar Rice factors because, at each sample point, they were subject to comparable scattering. The scattering for the facing channels, *h*_11_ and *h*_22_ channels were substantially different because they were spatially separated by four wavelengths. This, therefore, shows that the facing channels could be subject to significantly different multipath corresponding to different Rice factors up to 20. This warrants the need to characterize channels for different LAM environments with specific deployed antennas, all of which affect the Rice factor.

The Doppler spreads corresponding to each of the four measured channels are shown in figure 13*b*. Note that the *x*-axis plots frequency relative to the maximum Doppler shift, *f*_d|Max_, which ranges from ±6.25 *f*_d|Max_. As expected, the peak magnitude has zero Doppler shift and the Doppler components drop to a negligible level more than 20 dB down from the peak when greater than the maximum possible Doppler shift when *f*_d_ = ±*f*_d|Max_. It is difficult to create a Laplacian or Students-T filter model to fit this kind of Doppler spread accurately and as such a better approach is to generate the magnitude of the Rice fading using the well-known Classical Doppler filter [1] but to generate the phase variation differently, which will still result in modelling the same second-order statistics.

The narrow Doppler spread means that the Rice channel does not have a uniform phase distribution, which is generated in a LAM channel with the transmitter and receiver effectively stationary such that the line of sight path does not change but as the moving vessel transitions there are differing multipaths. This creates a phase characteristic, which depends on the number of paths in the channel at an instant. The Rice factor, *K*_f_, is derived by the power in the constant part (or line-of-sight) divided by the power in the non-constant part (or the scattered power) [1]. If there are E-fields arriving at the receiver from both the line-of-sight, *E*_LOS_, and *N*_Paths_ multipath rays, *E_n_*, with phase *ϕ*_LOS_ *ϕ_n_*, respectively, then the following two equations can be formed to define the Rice factor and the phase of the channel relative to a perfect line-of-sight, *ϕ*_LOS|Relative_:

5.1*a* $$K_{f}=\frac{{|E_{\mathrm{LOS}}|}^{2}}{\sum_{n}^{N_{\mathrm{Paths}}}{|E_{n}|}^{2}}$$

and

5.1*b* $$\phi_{\mathrm{LOS}|\mathrm{Relative}}=\arg\left[\frac{|E_{\mathrm{LOS}}|e^{j\phi_{\mathrm{LOS}}}}{|E_{\mathrm{LOS}}|e^{j\phi_{\mathrm{LOS}}}+\sum_{n=1}^{N_{\mathrm{Paths}}}|E_{n}|e^{j\phi_{n}}}\right].$$

**Figure 14.**
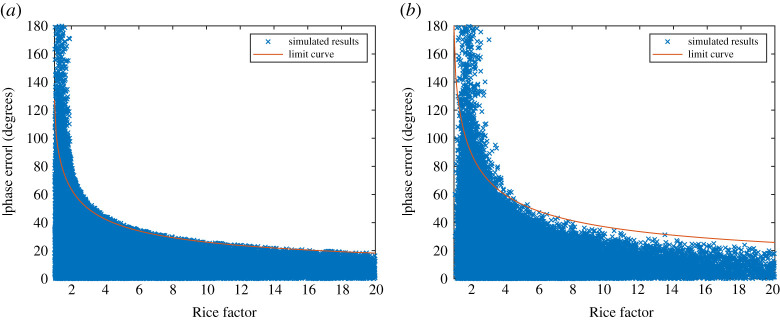
Monte Carlo simulations of |*ϕ*_LOS|Relative_| relative to Rice factor when (*a*) *N*_Paths_ = 2 and (*b*) *N*_Paths_ = 4. (Online version in colour.)

From equation (5.1*b*) it can be inferred that by increasing *N*_Paths_, there will be increased variation in *ϕ*_LOS|Relative_ while fewer paths will cause variation leading to zero in a perfect line-of-sight. The Rice factor will depend on the magnitude of the paths. In a LAM channel, with length typically less than 2 m, it is expected that *N*_Paths_ would be at least one from a ground reflection possibly with another path from another direction if the ground is cluttered, while the highest value is unlikely to exceed four. The actual number of paths would have to be evaluated for a given application and environment through extensive ray tracing and measurement. For this work, it will be assumed that *N*_Paths_ = 2 as a best case while *N*_Paths_ = 4 as a worst case. By inspection of equations (5.1*a*) and (5.1*b*), if it is assumed *E*_LOS_ is constant, then a Monte Carlo simulation can be run for random complex values of *E_n_*, which will result in a random value of *K*_f_ and *ϕ*_LOS|Relative_. This can determine the expected upper bound of *ϕ*_LOS|Relative_ for different values of *N*_Paths_. Figure 14*a* and *b* shows the simulated values of |*ϕ*_LOS|Relative_| against Rice factor for *N*_Paths_ = 2 and 4, respectively. A limit curve as a suitable curve of best fit to mark the maximum limit of simulated values where *K*_f_ > 6 is also plotted. In both cases, for low Rice factors below 6, the scattered power becomes significant in magnitude that it creates substantially deep fades close to that of a Rayleigh channel resulting in high values of |*ϕ*_LOS|Relative_|. Above this point, the phase has a more clearer limit that fits within the curve. Values from these curves can be stored in a lookup table to then define a limiting point at which the Rice channel can be generated with a given *K*_f_ and a random phase uniformly distributed with limited bounds of ±|*ϕ*_LOS|Relative_|.

### (b) Capacity analysis in the presence of multipath

**Figure 15.**
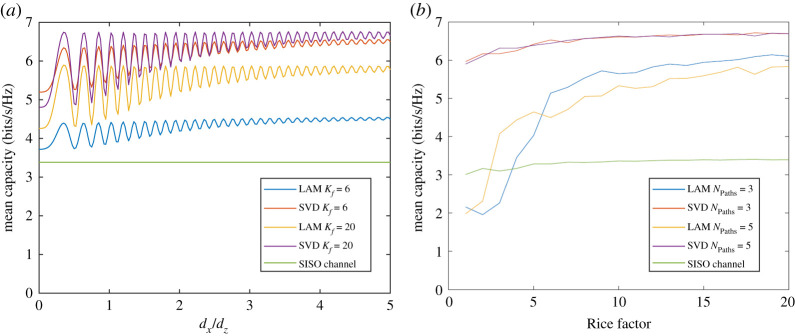
Simulations of LAM and SVD capacity in Ricean channels with 80^o^ element beamwidth showing (*a*) dependence on antenna separation for fixed Rice factor values and the assumption of *N*_Paths_ = 4 and (*b*) dependence on Rice factor for a fixed antenna separation of *d_x_*/*d_z_* = 5 with *N*_Paths_ = 3 and 5. (Online version in colour.)

Having established the channel model for both phase and magnitude, a new 2 × 2 Rice channel, **H**_Rice_, can be generated, which will form a new LAM multiplexing channel that exhibits fading: **H**_LAM_⊗**H**_Rice_ where ⊗ is the elementwise matrix multiplication function. The average capacity versus *d_x_*/*d_z_* for both LAM and SVD pre-coding is shown in figure 15*a* for random Ricean samples where *N*_Paths_ is fixed to 4, and the Rice factor is fixed at 6 and 20. Results are compared with the original SISO channel, and it is found that the average LAM capacity is over 80% of the limit when the spacing of *d_x_*/*d_z_* is greater than 3 when the Rice factor is 20. However, for a low Rice factor down to 6, the capacity drops substantially and is only as much as 30% above the SISO capacity.

To determine the impact of Rice factor and *N*_Paths_ on the average capacity, figure 15*b* plots the average capacity versus *K*_f_ when *d_x_*/*d_z_* is fixed at 5. Results are compared both for *N*_Paths_ = 3 and 5. Capacity with SVD pre-coding is not affected by *N*_Paths_ as it is essentially reconstructing the channel. LAM pre-coding on the other hand is still managing to achieve this sufficiently that *N*_Paths_ do not impact the result significantly. Clearly, it is the case that a Rice factor below 6 is not desirable for LAM multiplexing, while between 6 and 10, less than 85% of the maximum capacity is reached. Beyond this point, the capacity is near constant 85%, which provides a capacity gain of 1.7 times that of the SISO channel, therefore, 85% of the ideal case of double capacity. This factor of improvement can be scaled out to any size of *N*_x_ whereby the capacity gain would therefore be 0.85*N_x_*. For long moving, vessels values of *N_x_* could rise towards several hundred providing a substantially large spectral efficiency and low effort pre-coding.

## 6. Conclusion

The concept of LAM multiplexing has been presented in this paper and how it compares with OAM from which it has evolved. Results from synthetic channel analysis show the need for suitable antenna element spacing relative to the transmitter-to-receiver distance, which is dictated by the beamwidth of the antenna elements used. Channel measurements have been carried out for a worst case scenario with substantial clutter causing multipath, whereby a modified Ricean channel was found and the Rice factor ranges between 4 and 16. To implement LAM multiplexing for a given environment, it will require optimized antenna pattern design that will sustain connectivity to a moving vessel but also have suitable directivity to ensure a high Rice factor above 10 is achieved in order to reach the best available capacity limits. Such conditions would achieve spectral efficiencies at 85% of the capacity limit or more yet using substantially low effort pre-coding requiring little or no computation. The analysis presented in this paper shows LAM multiplexing as a primary means of having spectrally efficient wireless communications for moving vessels such as trains.

## Supplementary Material

Appendix

## Supplementary Material

LAM22 Meas

## Data accessibility

Data from the measurements in §5a used in this work are currently available in the submitted electronic supplementary material at rs.figshare.com.
