# Supplementary material for: Linear angular momentum multiplexing—conceptualization and experimental evaluation with antenna arrays
Source: Proc Math Phys Eng Sci. 2020 Oct 7;476(2242):20200209. doi: 10.1098/rspa.2020.0209 (PMC7655745; doi:10.1098/rspa.2020.0209)
Supplement: Appendix [file rspa20200209supp1.zip › Appendix_FinalSubmit.pdf]

PROCEEDINGS A

rspa.royalsocietypublishing.org

Research

Article submitted to journal

Linear Angular Momentum Multiplexing – Concept and Experimental Evaluation: Supplementary Material

Timothy D. Drysdale, Tim W. C. Brown, Ben H. Allen

This supplemental work considers Linear Angular Momentum (LAM) and Planar Angular Momentum (PAM) modes in a theoretical continuous form and derives the orthogonal modes in Euclidean space. The impact of phase error on mode detection is evaluated in terms of the resulting interference-to-signal ratio between the modes. The impact of Doppler shift on received wavefront angle is also evaluated.

THE ROYAL SOCIETY
PUBLISHING

© The Authors. Published by the Royal Society under the terms of the Creative Commons Attribution License http://creativecommons.org/licenses/by/4.0/, which permits unrestricted use, provided the original author and source are credited.

1. Linear and Planar Angular Momentum Modes

Planar Angular Momentum (PAM) modes represent the general class of modes which include the Linear Angular Momentum (LAM) modes that we introduce in the main paper. Analogous to Laguerre Gaussian modes[1], PAM modes rely on spatially-distributed phase patterns. PAM modes are equivalent to plane waves, with carefully chosen wave-vectors, $\vec{k}$, such that they are fully independent: when received in a given receiver aperture; under ideal conditions; and nearly independent when realistic imperfections are considered. We begin our analysis of PAM modes by considering the general case of a 2D receiving aperture that is stationary with respect to a transmitter, as shown in Figure 2. The transmitter can produce ideal plane waves in the receiving aperture, with arbitrary wave vectors[2]

$$\vec{k} = k_x\hat{\mathbf{x}} + k_y\hat{\mathbf{y}} + k_z\hat{\mathbf{z}} \tag{1.1}$$

where $\hat{\mathbf{x}}, \hat{\mathbf{y}}, \hat{\mathbf{z}}$ are the unit vectors in the x, y, z directions, and k_x, k_y, k_z are the magnitude of the wavevector $\vec{k}$ in each of those directions, such that the incoming plane waves seen by the receiver are tilted if desired, i.e.

$$k_x, k_y \geq 0. \tag{1.2}$$

Examples of the tilted wavefronts associated with an example sub-set of the possible LAM and PAM modes is shown in Figure 1. LAM modes are PAM modes with a tilt in only one direction, i.e. for LAM at least one of k_x, k_y must be zero, for the case where the transmitter and receiver are separated in the z direction.

The PAM mode number is a 2-tuple of mode numbers (l_x, l_y) representing the angles in the x–z and y–z planes that the wavevector makes with the inward facing normal of the receiver aperture, $\vec{r_{in}}$, scaled such that an integer is obtained if the total phase difference across the aperture in that plane is a multiple of 2π, i.e.,

$$(l_x, l_y) = \left(\frac{\psi(x_1, 0) - \psi(0,0)}{2\pi}, \frac{\psi(0, y_1) - \psi(0,0)}{2\pi} \right), \tag{1.3}$$

[1]Orbital Angular Momentum (OAM) modes are Laguerre Gaussian modes with a non-zero azimuthal parameter

[2]The wavelength λ is related to the magnitude of the wavevector $k_0 = \left|\vec{k}\right|$ as

$$\lambda = \frac{2\pi}{k_0}$$

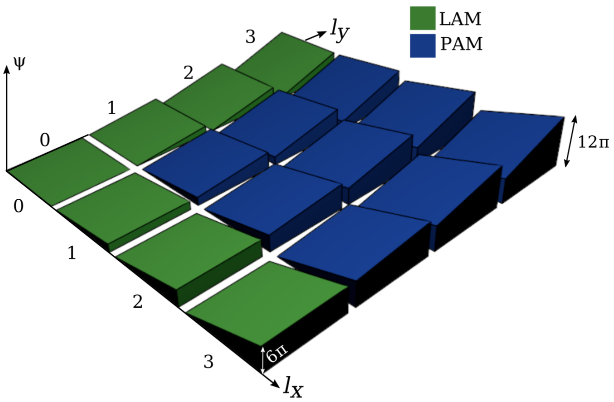

Figure 1: Wavefront tilts for a selection of LAM and PAM modes, where ψ on the vertical axis is the x, y-position-dependent phase of the mode, and l_x, l_y are the mode numbers in the x and y directions. Each tile represents a single mode.

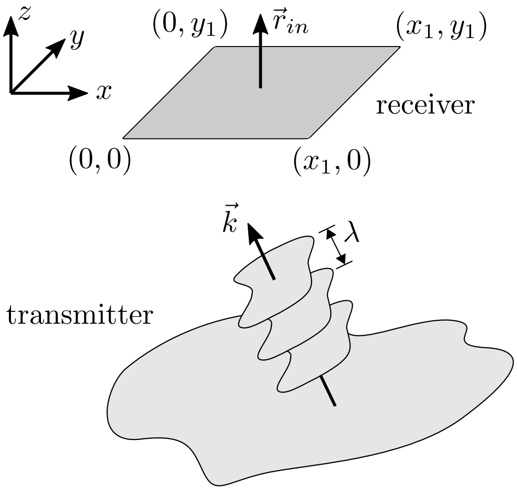

Figure 2: Diagram of a rectangular aperture receiving ideal plane waves with wavevector $\vec{k}$, and wavelength λ, from an arbitrary transmitter.

where $\psi(x, y)$ is the phase of the wave at the (x, y) position in the planar receiver aperture. The relationship between the mode number and the phase difference across the aperture is shown in Figure 3 for both the x–z and y–z plane. PAM modes with $lx, ly \in \mathbb{Z}$ are mutually independent, as we will now show.

2. Mode independence

We assume a planar rectangular receive aperature with sides aligned to the x, y directions, and opposite corners located at the points $(0, 0)$ and (x_1, y_1). The electric field E_t arising in the receive

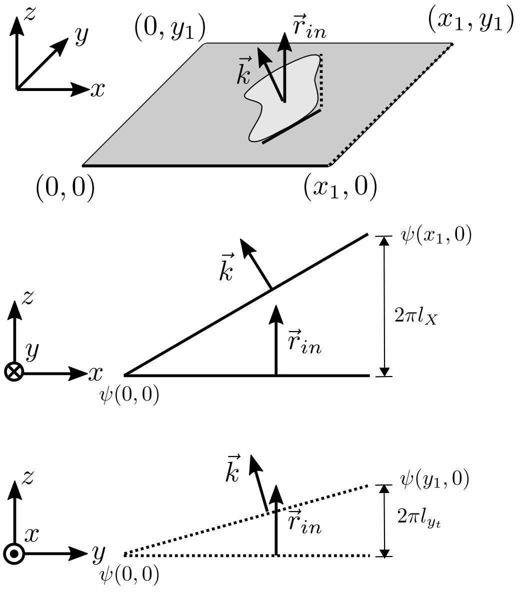

Figure 3: Diagram showing the PAM mode number calculation

aperture due to a transmitted PAM mode of mode number (l_{x_t}, l_{y_t}) is

$$E_t(x, y, l_{x_t}, l_{y_t}) = A_t e^{j\psi_t(x,y,l_{x_t},l_{y_t})} \tag{2.1}$$

where the amplitude A_t is constant across the aperture, but the phase distribution ψ_t is position dependent such that

$$\psi_t(x, y, l_{x_t}, l_{y_t}) = 2\pi \left(\frac{x}{x_1} l_{x_t} + \frac{y}{y_1} l_{y_t} \right). \tag{2.2}$$

In order to discriminate (receive) a particular mode (l_{x_r}, l_{y_r}) then the opposite phase shift ψ_r is applied across the aperture by the receiver

$$\psi_r(x, y, l_{x_r}, l_{y_r}) = -2\pi \left(\frac{x}{x_1} l_{x_r} + \frac{y}{y_1} l_{y_r} \right). \tag{2.3}$$

The normalised received signal amplitude A_r is given by

$$A_r(l_{x_t}, l_{y_t}, l_{x_r}, l_{y_r}) = \frac{1}{x_1 y_1} \int_0^{y_1} \int_0^{x_1} e^{j\psi_t(x,y,l_{x_t},l_{y_t})} e^{j\psi_r(x,y,l_{x_r},l_{y_r})} dx dy. \tag{2.4}$$

where we assume the amplitude of the transmitted modes are all $A_t = 1$. We note that the errors in the phase gradient can arise at either the transmitter, receiver, or both, but that errors in one are equivalent to the errors in the other. We can simplify our analysis, without loss of generality, to consider only the cross-talk between modes as a function of their mode-number differences $\Delta m, \Delta n$ in the x, y directions:

$$\Delta m = l_{x_t} - l_{x_r} \tag{2.5}$$

$$\Delta n = l_{y_t} - l_{y_r}. \tag{2.6}$$

$$\tag{2.7}$$

The received signal strength, A_r, is now expressible in terms of the mode differences Δm and Δn only. After re-arranging Eq. (2.4) and substituting Eqs. (2.5) & (2.6), A_r becomes [3]

$$A_r(\Delta m, \Delta n) = \frac{e^{j2\pi\Delta m} + e^{j2\pi\Delta n} - e^{j2\pi(\Delta m + \Delta n)} - 1}{4\pi^2 \Delta m \Delta n} \tag{2.8}$$

Equation (2.8) immediately shows that PAM modes are completely independent ($A_r = 0$) so long as the mode numbers are strictly integer, and the mode differences in each direction are both integers greater than zero. For the case that the mode difference is zero in either or both directions, the bottom line goes to zero, requiring further analysis. For the case that both mode differences are zero, it is desired that the received signal is at a maximum (because the desired mode is being transmitted). In such a case, l'Hopital's rule can be used to show that the mode is indeed received at maximum strength as desired. Let $x = \Delta m = \Delta n$ then

$$\begin{aligned} A_r(0,0) &= \lim_{x \to 0} \frac{f(x)}{g(x)} \\ &= \lim_{x \to 0} \frac{f'(x)}{g'(x)} \\ &= \lim_{x \to 0} \frac{f''(x)}{g''(x)} \\ &= \lim_{x \to 0} \frac{-8\pi^2 \left(e^{j2\pi x} - 2e^{j4\pi x} \right)}{8\pi^2} \\ &= 1 \end{aligned}$$

[3]PAM mode numbers are defined with respect to the receiver aperture size, so the mode independence calculated for one receiver does not necessarily apply to another, unless they have the same aperture dimensions. The influence of the aperture dimensions are still included in Eq. (2.8) but they are no longer explicit - they are subsumed into the values of Δm and Δn.

For the case where the mode difference is zero in only one direction, such as is the case for all undesired LAM modes, and some undesired PAM modes, then the received signal strength

$$A_r(\Delta m, 0) = A_r(0, \Delta n) = A_r(\Delta l) \tag{2.9}$$

where $\Delta l = \Delta m$ or $\Delta l = \Delta n$ depending on whether the mode difference is in the x- or y-direction, and

$$A_r(\Delta l) = \frac{\mathrm{j}\left(1 - e^{\mathrm{j}2\pi\Delta l}\right)}{2\pi\Delta l}. \tag{2.10}$$

The sensitivity to mode differences in one direction is the same, regardless of whether that difference is in the x- or y-direction. After some manipulation, it is possible to show that the magnitude of the received signal is

$$|A_r(\Delta l)| = \mathrm{sinc}(\pi\Delta l). \tag{2.11}$$

We summarise the performance of PAM in Table 1

3. LAM

The analysis of mode independence in the previous section addressed LAM modes in Eq. (2.11), because for LAM, the mode difference in one direction is always zero, by definition. Equation (2.11) highlights that integer LAM modes are independent as expected ($A_r(\Delta l) = 0$ for $\Delta l \in (Z)^+$), but that practical systems can also tolerate small variations in the mode number without losing reception, i.e. the signal to noise ratio remains sufficient to attain the required signal demodulation performance. On the other hand, the usage of fractional mode numbers where there are modes with spacing of less than one mode number will lead to increased crosstalk. We define the Interference-to-Signal Ratio (ISR) so that we can evaluate the impact of any individual undesired mode's crosstalk on the desired received signal as

$$\mathrm{ISR} = \frac{A(\Delta l)}{A(0,0)} \tag{3.1}$$

Equation (3.1) is calculated numerically using Eq. 2.11 and plotted in Figure 4. The inset in Figure 4 shows that for adjacent modes, if the error in producing the mode with the correct gradient can be kept to a mode number difference of less than three percentage points (i.e. $\Delta l \leq 0.03$) from the expected value then the interference from that mode will be less than -30dB.

(a) Aperture selection

The receiver aperture size constrains the set of the angles at which integer mode numbers occur. The angles of the planes of constant phase are plotted in Figure 5 for representative cases where the aperture size x_1 is set to values $\lambda \leq x_i \leq 10\lambda$, and for modes up to $l_{x_t} \leq 10$. For smaller apertures, the angular spacing between modes reduces dramatically as the mode number increases, reducing below $3°$ for modes $l \geq 4$, as shown in Figure 6. Carrying over the $\Delta l \leq 0.03$

Table 1: Received signal amplitude for PAM modes as a function of mode differences

case	$\lvert A_r\rvert$	comment
$\Delta m = \Delta n = 0$	1	desired mode received
$\Delta m \in \mathbb{Z}^+, \Delta n = 0$	0	undesired integer mode blocked
$\Delta m = 0, \Delta n \in \mathbb{Z}^+$	0	undesired integer mode blocked
$\Delta m, \Delta n \in \mathbb{Z}^+$	0	undesired integer mode blocked
$\Delta m \in \mathbb{R}, \Delta n = 0$	$\mathrm{sinc}(\pi\Delta m)$	undesired fractional mode causes crosstalk
$\Delta m = 0, \Delta n \in \mathbb{R}$	$\mathrm{sinc}(\pi\Delta n)$	undesired fractional mode causes crosstalk
$\Delta m, \Delta n \in \mathbb{R}$	see Eq.(2.8)	undesired fractional mode causes crosstalk

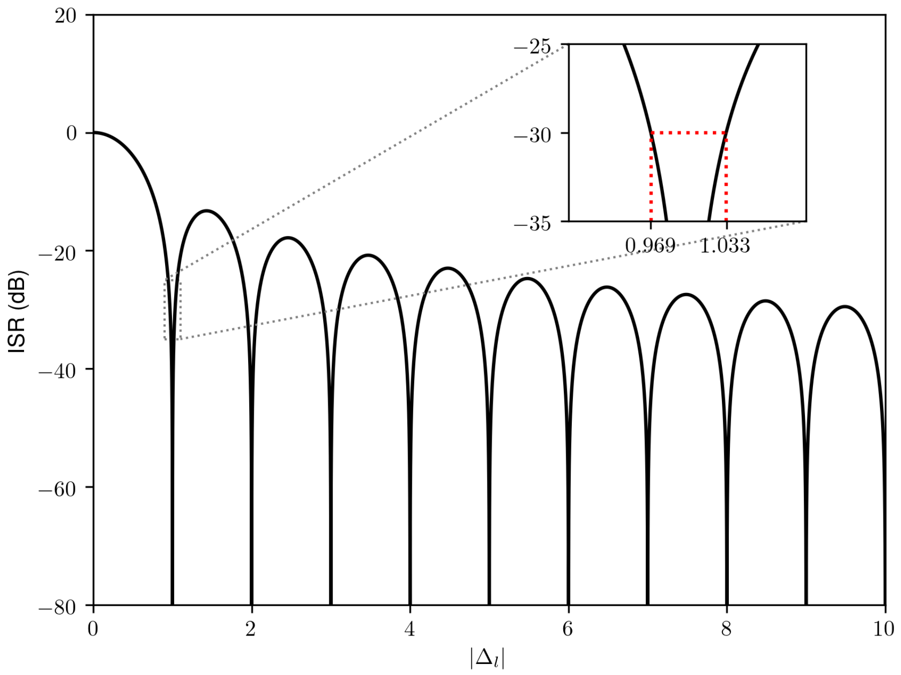

Figure 4: Log plot of the interference strength of an unwanted mode that is Δl mode numbers away from the desired mode

mode number tolerance from the previous section, we can calculate the maximum deviation from the desired wavefront angle, as shown in Figure 7. This shows that precise control of phase is essential in multimode systems. Note that these plots have omitted negative mode numbers for convenience, thus system implementers can double the number of modes available for a given tolerance level as compared to the data plotted in Figure 7.

(b) Doppler shift

The application domain of PAM and LAM wireless communications potentially includes fast moving vehicles, but we evaluate the phase-error introduced by Doppler shifts to be negligible. The rest of the section describes a calculation of the expected shift using the Lorentz boost. We consider a planewave with angular frequency ω_0 and wave-vector $\vec{k_0}$ in the reference frame K' in which the source is at rest, such that the angle it makes with the x' axis is θ_0, where the subscript zero indicates values in the frame where the source is at rest. An observer is travelling in the positive x-direction (parallel to the x' direction) at a speed of $\vec{v} = 300\,\mathrm{m\,s^{-1}}\hat{\mathbf{x}}$ ($v = 1080\,\mathrm{km\,h^{-1}}$) in reference frame K, which is similar to the fastest speeds obtained by ground vehicles to date. Using an inverse Lorentz transformation it can be shown [1] that the familiar Doppler shift is

$$\omega = \gamma\omega_0(1 + \frac{k_0}{\omega_0} v \cos\theta_0) \tag{3.2}$$

where γ is the Lorentz factor

$$\gamma = \sqrt{1 - \frac{v^2}{c^2}} \tag{3.3}$$

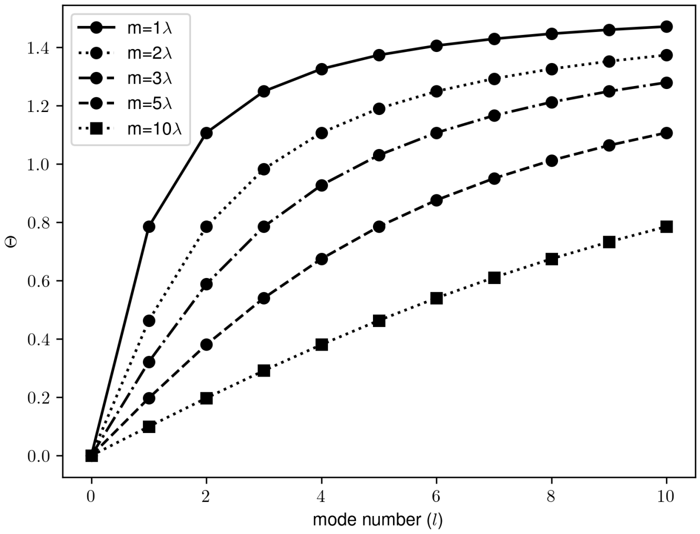

Figure 5: Angles of the PAM modes (plane waves) as a function of mode number and aperture size

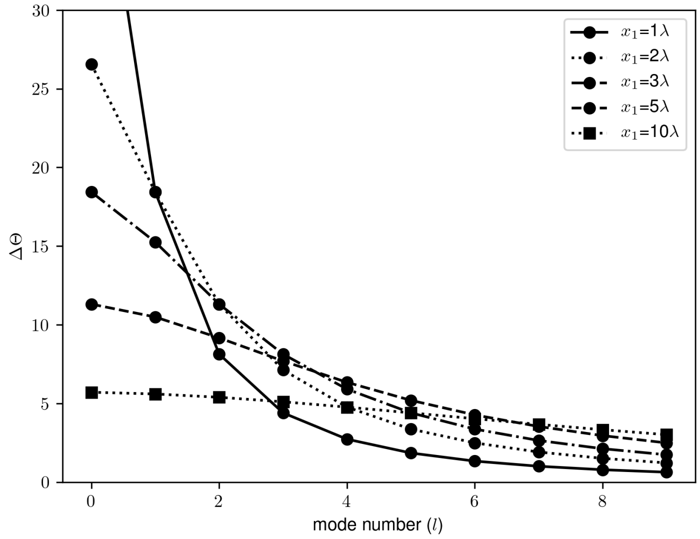

Figure 6: Angular spacing between adjacent PAM modes a function of mode number and aperture size

and the speed of light is $c \approx 3 \times 10^8 \mathrm{m\,s}^{-1}$. For a $1\,\mathrm{GHz}$ signal, the Doppler shift amounts to less than $300\,\mathrm{Hz}$. Note that there is still a non-zero Doppler shift for the $l_{x_t} = 0$ mode with wave-vector perpendicular to the direction of travel, albeit not a significant one. The phase gradient is also potentially affected, because k_x in the observer's frame K is transformed from k_{0_x} in the

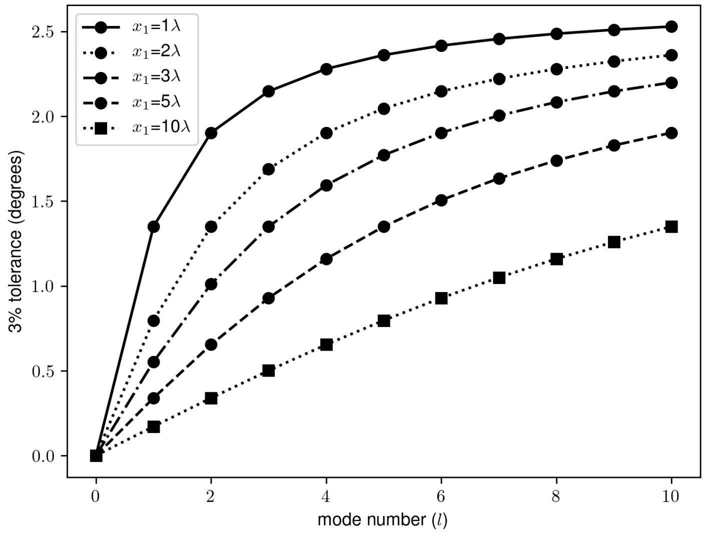

Figure 7: Angular tolerance arising from the 3% mode tolerance that achieves less than -30dB crosstalk

source rest frame K' due to the relative motion, with the new angle of the wavefront θ being expressible [1] as

$$\theta = \arctan \frac{\sin\theta_0}{\gamma\left(\cos\theta_0 + v(\omega_0/k_0)/c^2\right)}. \tag{3.4}$$

The absolute difference in perceived mode number between the two reference frames is plotted in Figure 8 for a range of aperture sizes and modes. All the values plotted are below 8.2×10^{-5}, which indicates any perceived difference in the mode number as seen by the observer, will be so small as to be undetectable in any practical implementation. Therefore, the influence of Doppler shifts can be neglected without hazard, at least for vehicles travelling at typical Newtonian speeds.

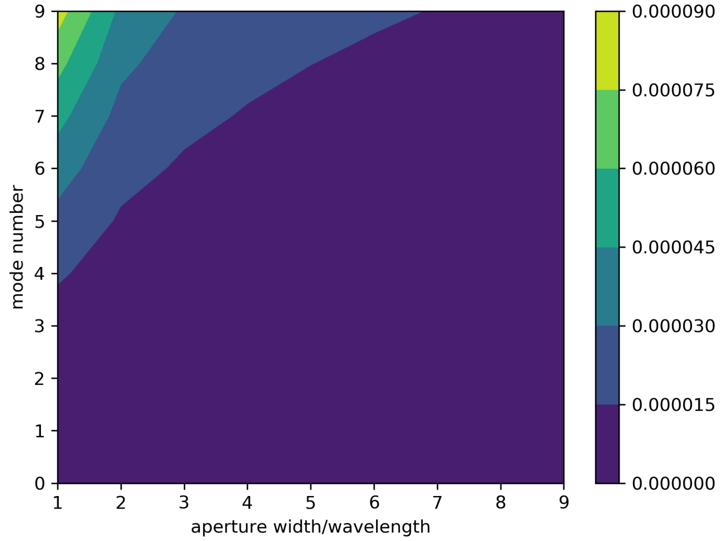

Figure 8: Difference in perceived mode number due to Doppler shift, for a 1GHz signal when vehicle is travelling at 1080km/h

References

1. Steane AM. 2012 *Relativity Made Relatively Easy*. Oxford, United Kingdom: Oxford University Press.
