# Supplementary figures and images for: Linear angular momentum multiplexing—conceptualization and experimental evaluation with antenna arrays
Source: Proc Math Phys Eng Sci. 2020 Oct 7;476(2242):20200209. doi: 10.1098/rspa.2020.0209 (PMC7655745; doi:10.1098/rspa.2020.0209)

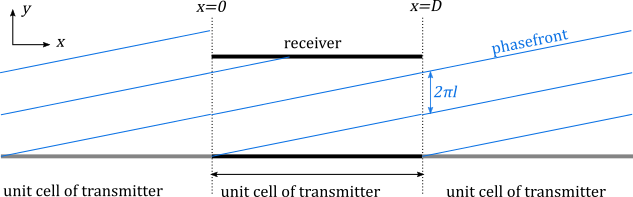

Supplement: Appendix [file rspa20200209supp1.zip › geometry.png]

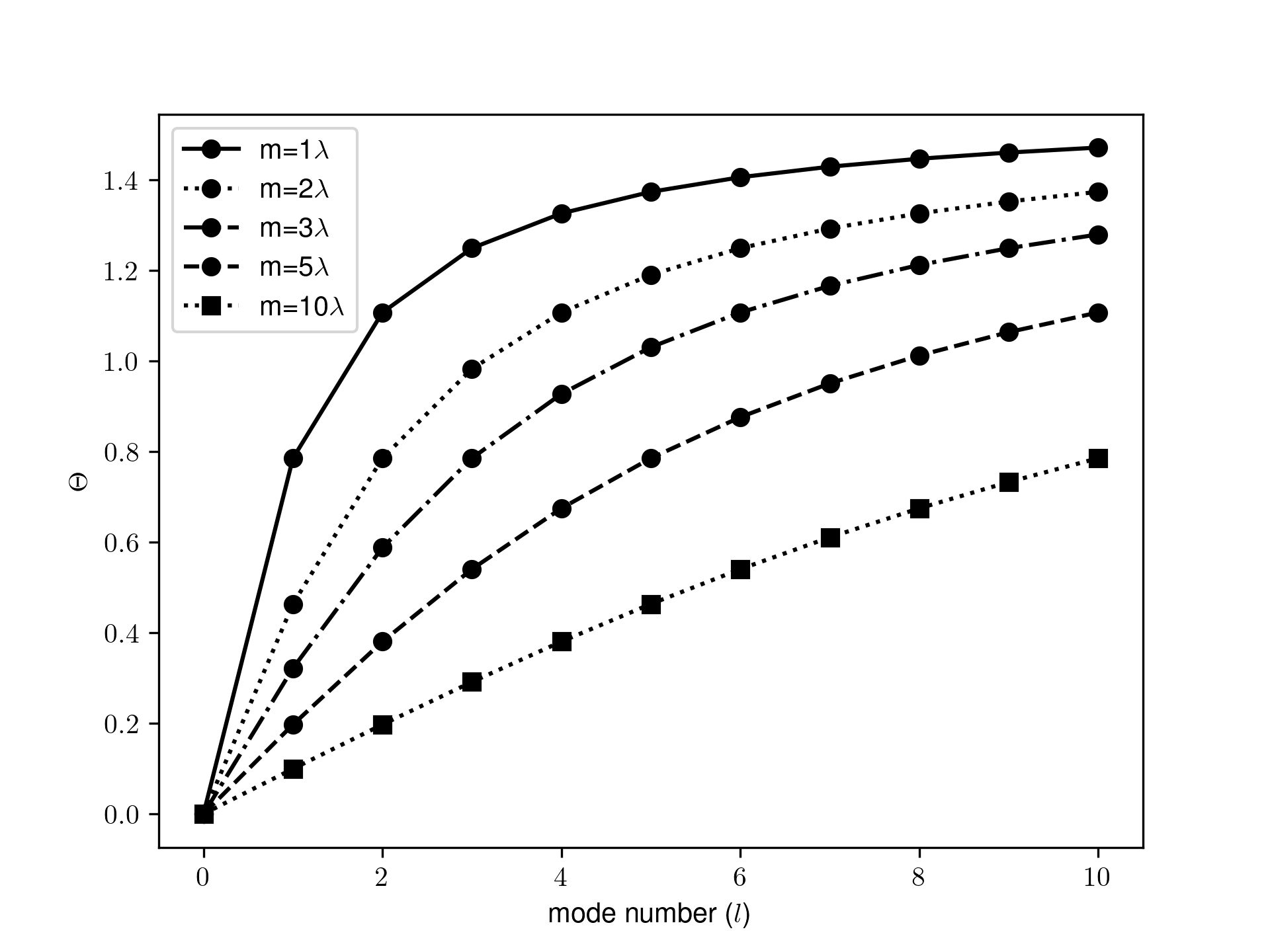

Supplement: Appendix [file rspa20200209supp1.zip › LAM_angles.png]

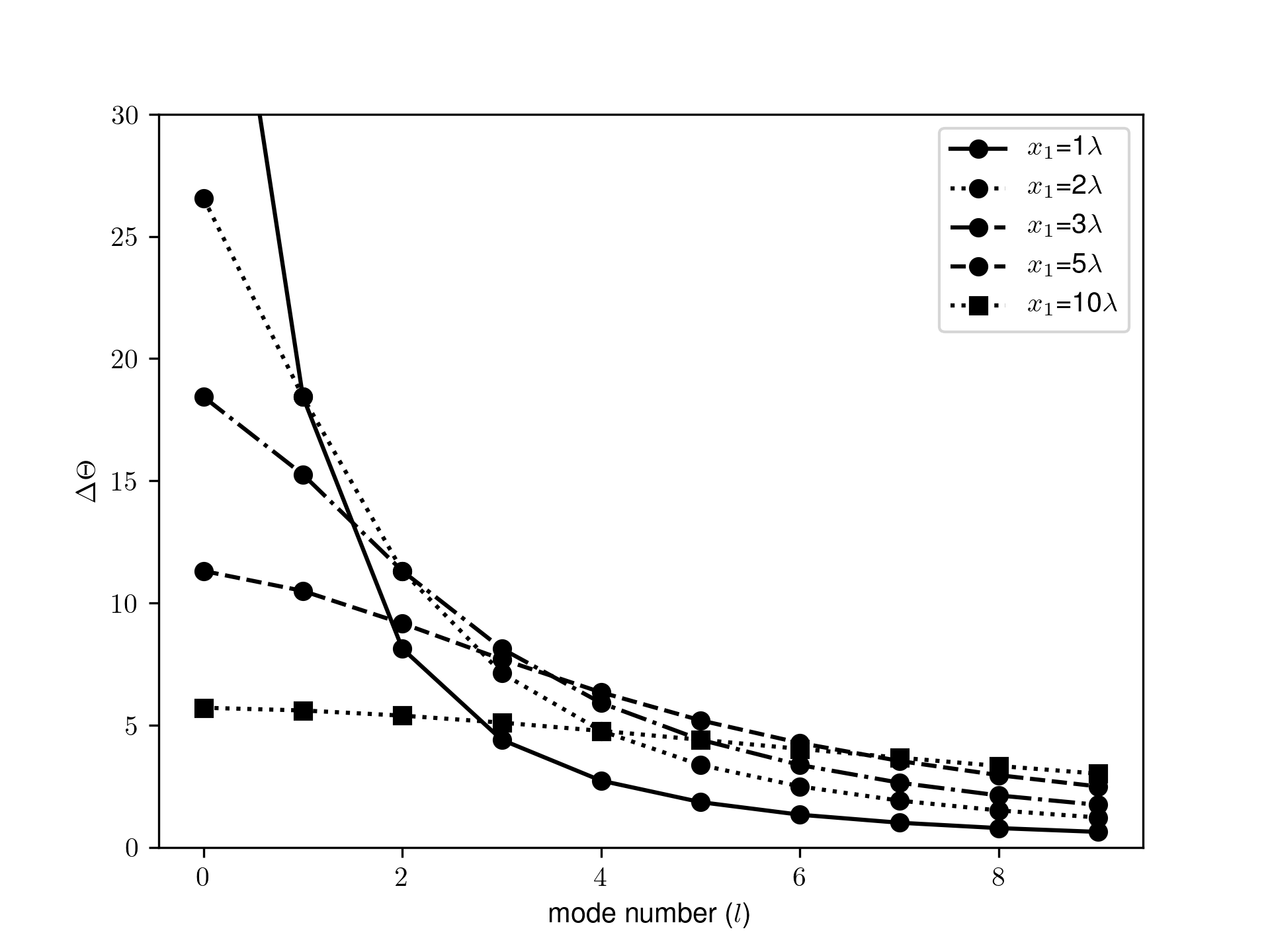

Supplement: Appendix [file rspa20200209supp1.zip › LAM_angles_diff.png]

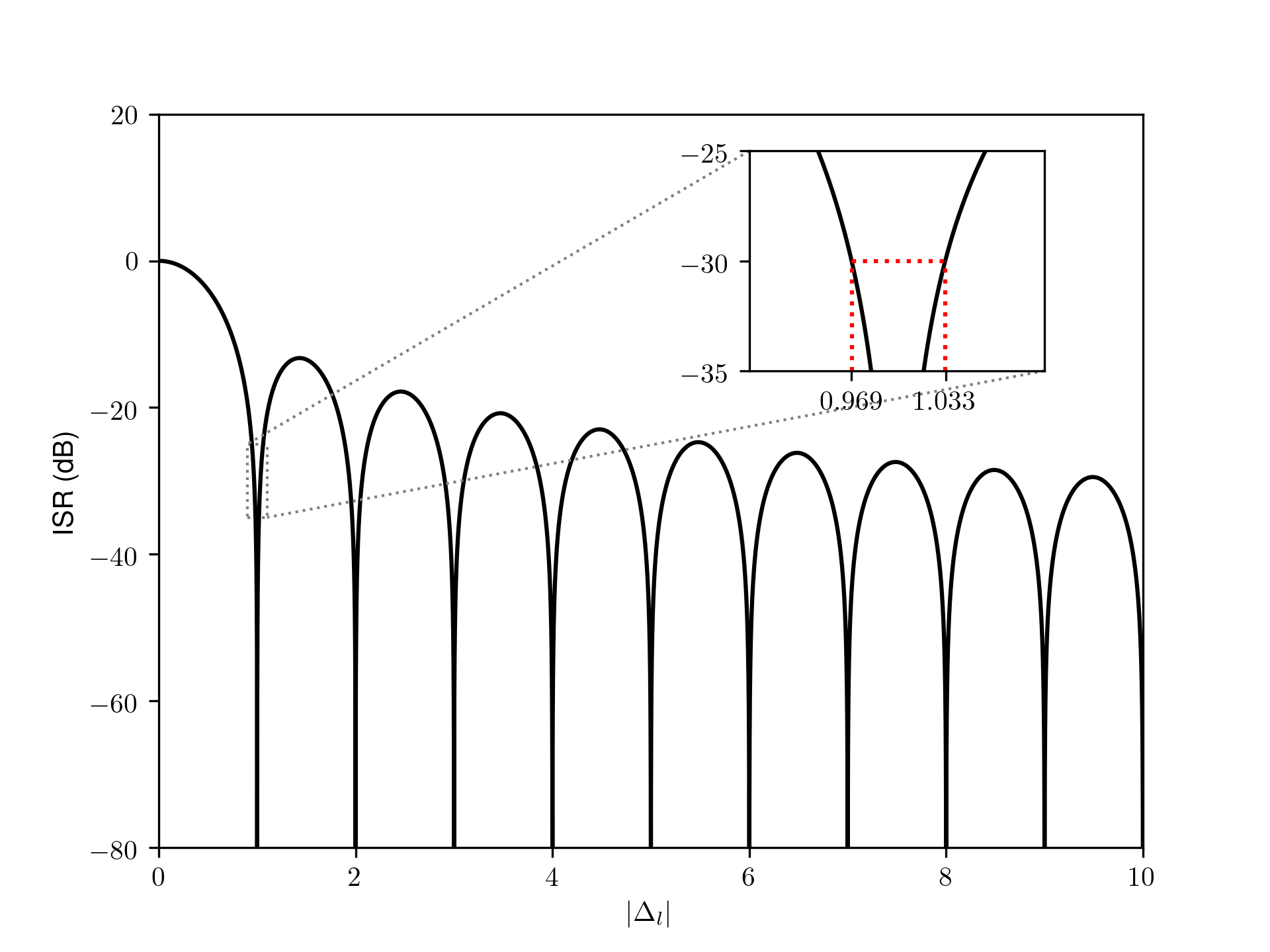

Supplement: Appendix [file rspa20200209supp1.zip › LAM_ISR_dB.png]

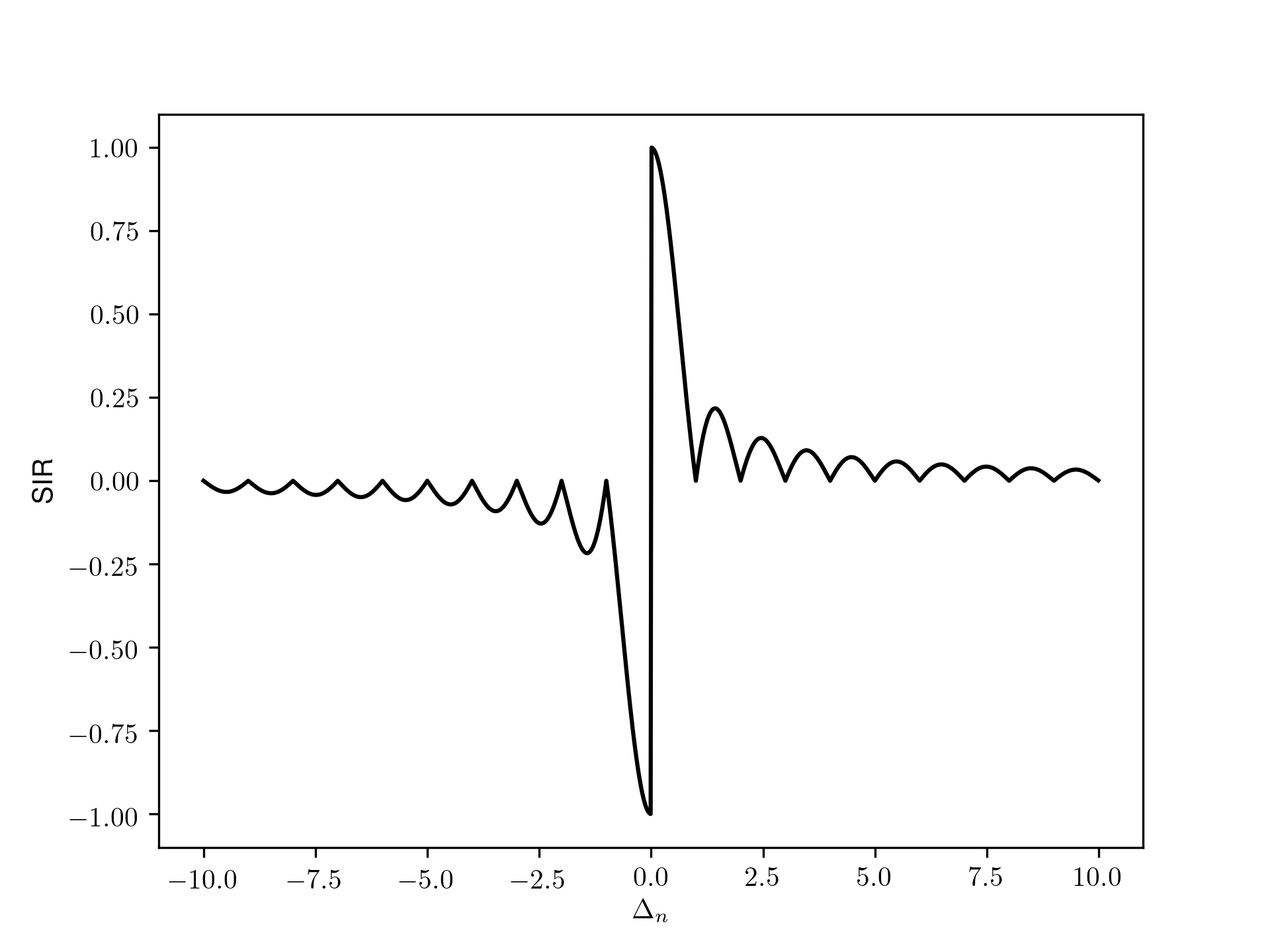

Supplement: Appendix [file rspa20200209supp1.zip › LAM_SIR.png]

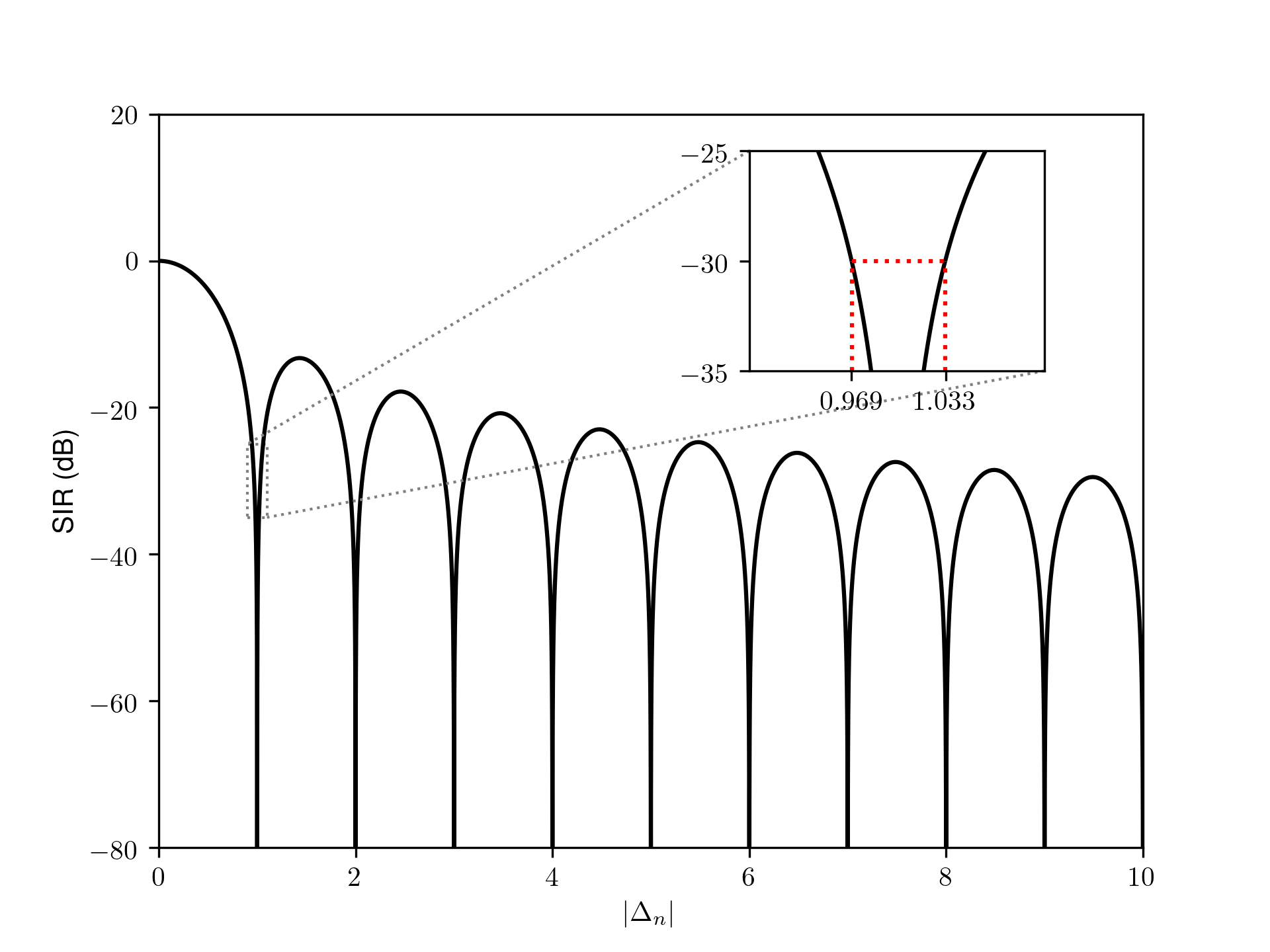

Supplement: Appendix [file rspa20200209supp1.zip › LAM_SIR_dB.png]

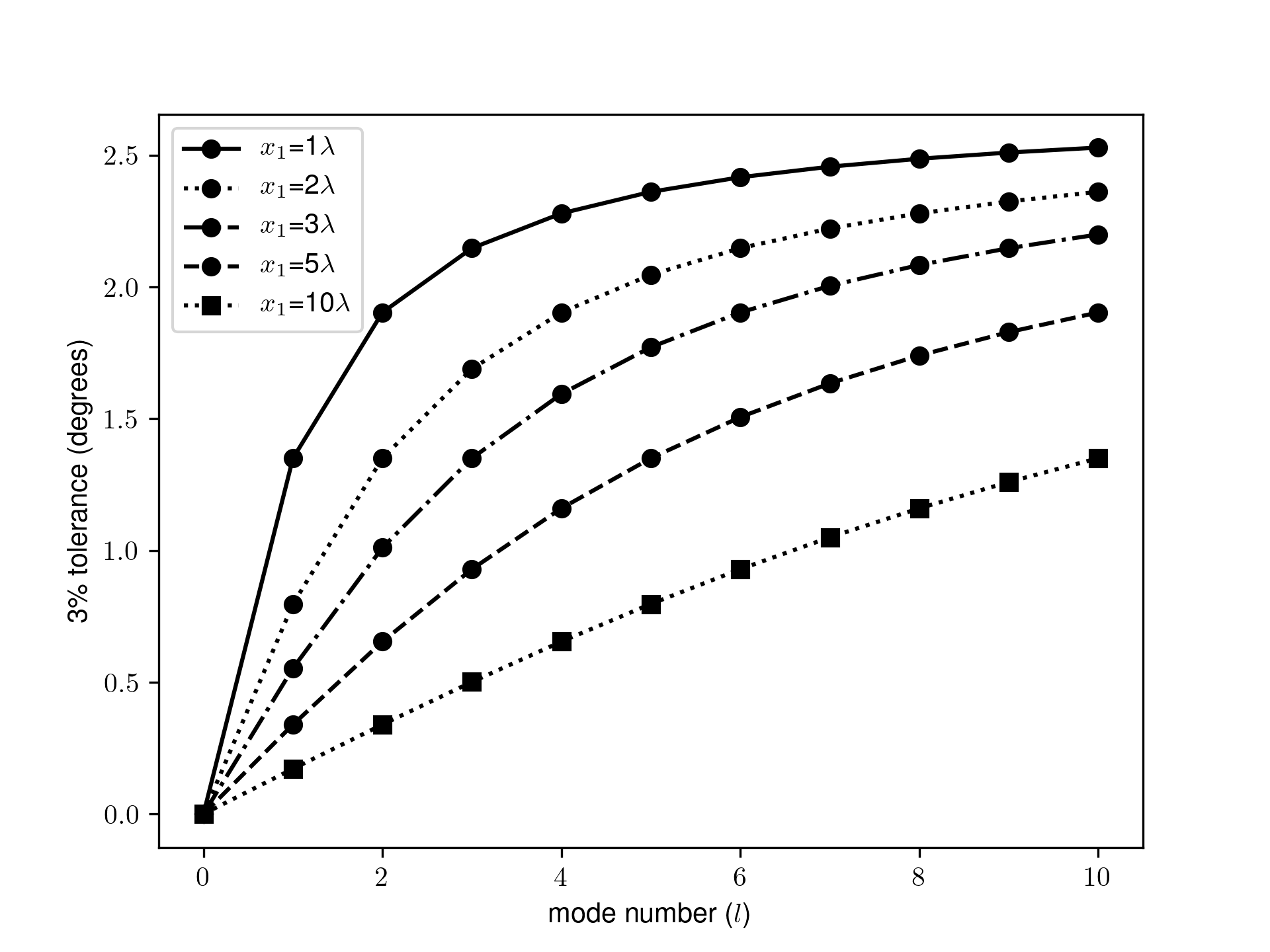

Supplement: Appendix [file rspa20200209supp1.zip › LAM_tolerance.png]

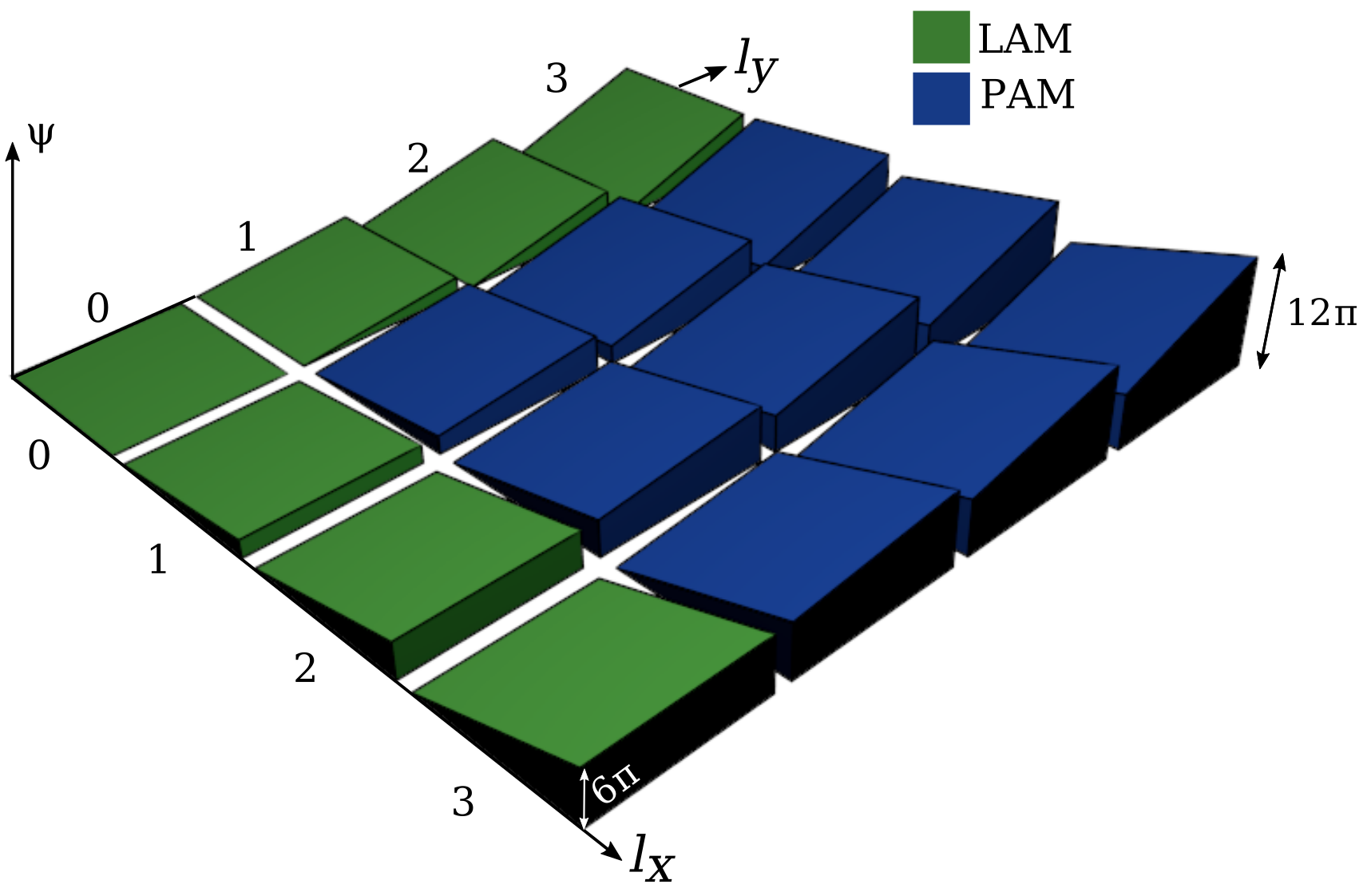

Supplement: Appendix [file rspa20200209supp1.zip › lampam.png]

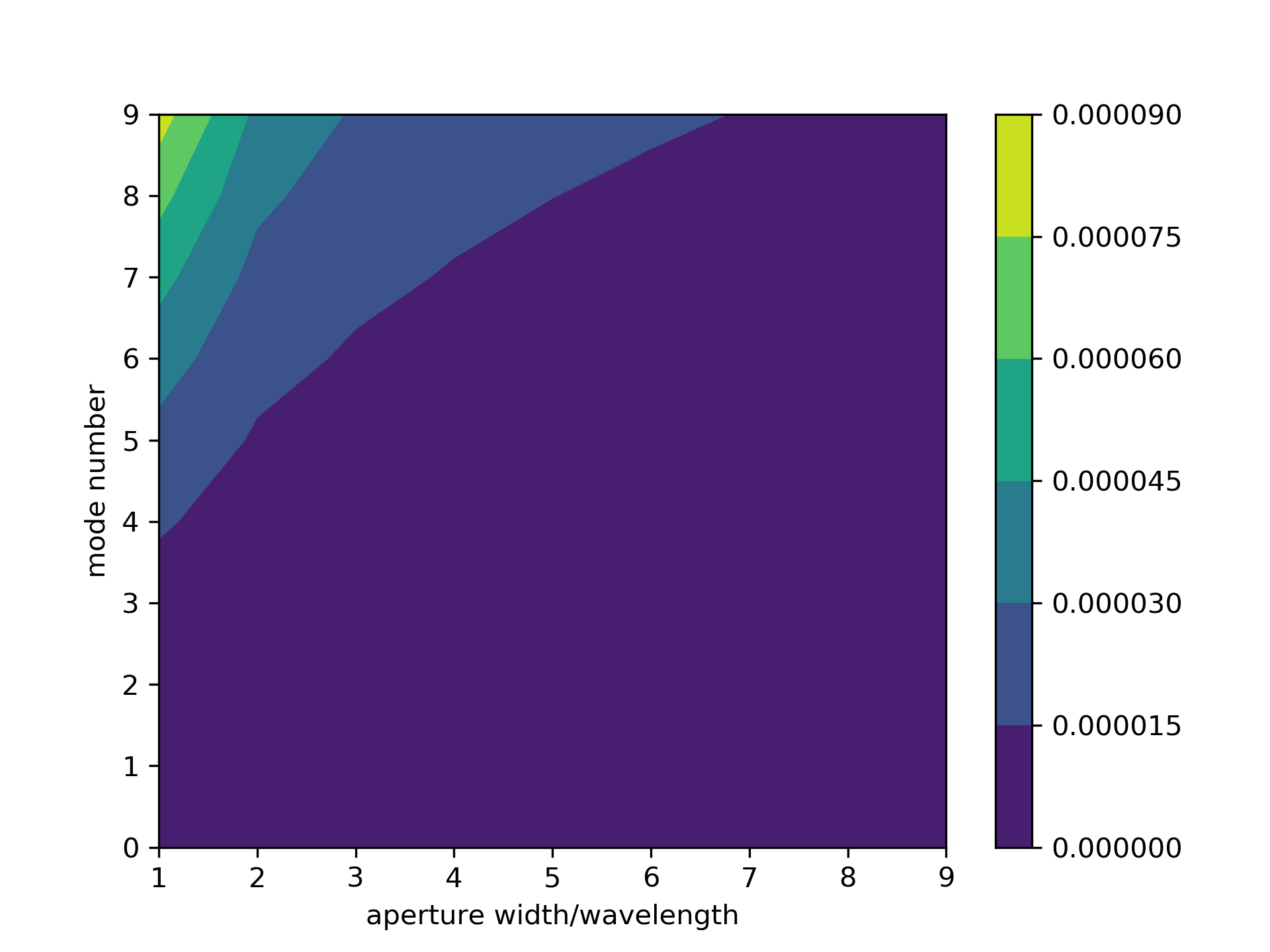

Supplement: Appendix [file rspa20200209supp1.zip › modenumberdifference.png]
